# Self-reported prevalence and potential factors influencing cardio-cerebral vascular disease among the Chinese elderly: A national cross-sectional study

**DOI:** 10.3389/fcvm.2022.979015

**Published:** 2022-10-20

**Authors:** Lingbing Meng, Jiapei Xu, Jianyi Li, Jiabin Hu, Hongxuan Xu, Dishan Wu, Xing Hu, Xuezhai Zeng, Qiuxia Zhang, Juan Li, Tao Gong, Deping Liu

**Affiliations:** ^1^Department of Cardiology, Beijing Hospital, National Center of Gerontology, Institute of Geriatric Medicine, Chinese Academy of Medical Sciences, Beijing, China; ^2^Graduate School, Chinese Academy of Medical Sciences and Peking Union Medical College, Beijing, China; ^3^Graduate School, Peking University Fifth School of Clinical Medicine (Beijing Hospital), Beijing, China; ^4^Health Service Department of the Guard Bureau of the Joint Staff Department, Beijing, China; ^5^China Research Center on Aging, Beijing, China; ^6^Center on Aging Psychology, Key Laboratory of Mental Health, Institute of Psychology, Chinese Academy of Sciences, Beijing, China; ^7^State Key Laboratory of Brain and Cognitive Science, Institute of Biophysics, Chinese Academy of Sciences, Beijing, China; ^8^Department of Neurology, Beijing Hospital, National Center of Gerontology, Institute of Geriatric Medicine, Chinese Academy of Medical Sciences, Beijing, China

**Keywords:** CCVD, SSAPUR, elderly, multivariate linear regression model, risk factors

## Abstract

**Background:**

Aging is an essential national condition throughout China in the 21st century. Cardio-cerebral vascular disease (CCVD) is a common chronic vascular disease in the elderly. Despite aging becoming an increasingly pressing issue, there has been no comprehensive national investigation into the risk factors, prevalence, and management of CCVD among the elderly population in China.

**Materials and methods:**

Through the 4th Survey of the Aged Population in Urban and Rural China (SSAPUR), a nationally representative sample of 224,142 adults aged more than 60 years was surveyed using a multistage, stratified sampling method. The 4th SSAPUR was used to investigate CCVD in the elderly. Univariate and multivariate logistic proportional regression analyses explored the risk factors. These risk factors were then entered into a multivariate linear regression model to identify independent predictive factors for CCVD. Disease management was assessed from the self-reported history of physician diagnosis, treatments, and hospital visits among individuals with CCVD.

**Results:**

After excluding samples with missing information, 215,041 individuals were included in the analysis. The overall prevalence of CCVD was 26%. Living in a rural area, being older, being female, having low literacy, smoking, getting little sleep, losing a spouse, being single, not getting enough exercise, having a bad financial situation, and not taking part in public welfare programs were the main risk factors for CCVD among the elderly in China (*P* < 0.05). In the multivariate linear regression model, holding all other variables at any fixed value, CCVD remained associated with “urban and rural” (β = 0.012, *P* < 0.001), “age” (β = −0.003, *P* < 0.001), “sex” (β = −0.022, *P* < 0.001), “education level” (β = −0.017, *P* < 0.001), “marriage” (β = 0.004, *P* = 0.047), “smoking” (β = 0.012, *P* = 0.003), “drinking” (β = −0.015, *P* = 0.001), and “sleep” (β = 0.008, *P* = 0.005). There were no collinearity problems among these factors.

**Conclusion:**

Major risk factors for prevalent CCVD among the elderly in China include the following: rural residence, female, low literacy level, poor sleep quality, bereavement, non-marriage, living alone, lack of exercise, poor financial situation, and non-participation in public welfare activities. Chinese national policies for preventing, controlling, and managing risk factors for CCVD in the elderly must be urgently developed.

## Introduction

An aging population is an essential national condition throughout China in the 21st century (1). By the end of 2014, the number of people aged 60 years or older in China had reached 212 million, accounting for 15.5% of the total population. According to the National Strategy Research Group of the Office of the China Working Commission on Aging, the number of older adults in China will peak at 487 million by 2053, accounting for 34.9% of the total population. This rapidly growing aging population will exert a long-term and profound impact on China’s economic, political, cultural, social, and ecological progress (2). China’s long-term strategic task is to deal with this aging population actively. To do so will require decision-making based on scientific evidence. The first premise of scientific decision-making is to carry out comprehensive and in-depth investigations and research.

Cardio-cerebral vascular disease (CCVD) is a common chronic vascular disease in the elderly. The death toll caused by CCVD accounts for about 40.27% of the total mortality in the country. According to current trends, by 2030, the prevalence of coronary heart disease in China will have increased by 3.7 times compared with 2000 (3). According to the 2018 China Cardiovascular Disease Report, there are 290 million patients with CCVD in China, including 13 million with stroke and 11 million with coronary heart disease. The Stroke Screening Project showed that, in 2014, the prevalence of stroke among adults older than 40 years in China was 2.06%. Strokes can be divided into hemorrhagic strokes, caused by a sudden rupture of a blood vessel in the brain, and ischemic strokes, caused by a blockage of a blood vessel, which is opposed to each other. The subtypes of stroke are intracerebral hemorrhage, subarachnoid hemorrhage, and cerebral ischemia. The mortality rate due to coronary heart disease was higher among men than women (4). Smoking dramatically increases people’s risk of other CCVD events (5). The incidence of CCVD increases with population changes. Age remains the most significant irreversible risk factor for CCVD. However, modifiable factors related to diet, lifestyle, physical activity, and psychosocial conditions also play a key role in the occurrence and development of CCVD (6).

An analysis of 31,343 elderly individuals published in 2017 pointed out that old-age frailty is closely related to CCVD and constitutes a changeable risk factor for CCVD in the elderly. Old-age frailty increases the risk of CCVD (7). There is considerable evidence to suggest that aging is an actual new disease in the 21st century and that it plays a vital role in developing CCVD (8).

We conducted the 4th Survey of the Aged Population in Urban and Rural China (SSAPUR) among a large, nationally representative sample of elderly Chinese individuals aged 60 years or more (9, 10) to estimate the prevalence of CCVD in this age group. Furthermore, we researched risk factors for prevalent CCVD and documented the current management of CCVD among the Chinese elderly. The purpose of our study was to provide vital data necessary to decrease the prevalence rate of CCVD among the elderly in China. Chinese national policies for preventing, controlling, and managing risk factors for CCVD in the elderly must be urgently developed.

## Materials and methods

### Study participants

This study involved an extensive survey of a large sample (224,142) of elderly, nationally representative Chinese individuals (≥60 years old, who were permanent residents on August 1, 2015) by using the 4th SSAPUR. The number of valid questionnaires obtained was 215,041. The same inclusion criteria were used to find a replacement for those elderly individuals who were lost to follow-up or declined to participate in the survey. This survey began on August 1, 2015, and lasted for 1 month until 31 August. The China National Committee on Aging conducts the SSAPUR. It is a follow-up investigation with a large sample size covering all provinces of China. The survey includes questions about population, economy, health, services, social participation, spiritual culture, rights protection, livable environment, and many other aspects.

The survey adopts multi-level sampling and tracking methods, selecting Chinese citizens over 60 years as the survey participants. The simple sampling idea is as follows: First, we determine the sampling ratio, 1/1000. Secondly, the sample number shall be allocated according to the proportion of the elderly population in each province in the whole country. After that, we determine the sampling number of counties, towns, and communities and select 462 counties. According to PPS sampling, select four towns from each county and four communities (village or neighborhood committee) from each township.

At last, equidistant sampling was used to select 30 older adults from each community. A large variety of data has been collected through household interviews and questionnaire surveys. In China, SSAPUR is by far the largest database of older adults. This study used data from the 4th SSAPUR, conducted in 2015. The 4th SSAPUR achieved for the first time the goal of a nationwide survey, covering all provinces, autonomous regions, municipalities, and Xinjiang Production and Construction Corps across the country, involving 466 counties (districts), 1,864 townships (sub-districts), and 7,456 villages (residential) committees. A multistage stratified cluster sampling procedure was utilized, and the final stage involved equal probability sampling. More details of the 4th SSAPUR study design and the sampling method are described in the Supplementary Materials 1, 2.

We selected 31 provinces/autonomous regions/municipalities directly under the Central Government/Xinjiang Production and Construction Corps. The survey achieved the goal of covering the entire country for the first time, representing all of China’s socioeconomic conditions and lifestyles. The research protocol was approved by the Ethical Review Committee of Beijing Hospital (2021BJYYEC-294-01) and the National Bureau of Statistics (No. [2014] 87). All participants provided written informed consent.

### Procedures

We used the 4th SSAPUR questionnaire, which has been used previously in large-scale epidemiology studies, including the Global Burden of Disease study and the World Health Survey.

Age, sex, education level, marriage, living alone, smoking, drinking, sleeping, exercising, hypertension, diabetes, stomach diseases, medical insurance, whether other older adults in the home require care, whether they are working for a living, economic status, lack of involvement in public welfare programs, the absence of external abuse, and non-religious cultural life were among the data collected.

### Outcomes

We defined CCVD based on self-reported CCVD diagnosis by a physician using the 4th SSAPUR questionnaire, which has been validated and used by several regional CCVD surveys in China and was further validated by our in-house study. China’s Law on the Protection of Rights and Interests of the Elderly stipulates that the starting age of the elderly is 60 years old. Every citizen of the People’s Republic of China who has reached 60 years of age belongs to the elderly group.

### Statistical analysis

All calculations were weighted to represent the general adult population aged 60 years or more in China, according to the Fourth Sampling Survey on the Living Conditions of the Elderly Population in Urban and Rural China (2015), and sampling clusters stratified the results. We calculated the weightings using data from the CPH study and the study sampling scheme. Age-standardized prevalence of CCVD was calculated based on the 4th SSAPUR study (2015). The absolute number of people with CCVD was calculated based on the 2015 Chinese elderly population. Our analysis included 215,041 participants for whom the variables of interest were available. From a total of 223,680 cases, we deleted those with missing data, including 7,121 cases whose CCVD status was unclear and 17 individuals for whom more than ten independent variables were missing.

We assessed the statistical significance of differences using a χ^2^-test for categorical variables and a *post hoc* two-tailed Newman–Keuls test when three groups were compared. The relationship between risk variables and CCVD was next studied using single- and multivariate logistic proportional regression models. These risk factors were then entered into a multivariate linear regression model to identify independent predictive factors for CCVD. As the outcome of CCVD events (yes or no) is a categorical variable, it was assigned and coded with Dummy Regression before linear regression analysis. The coded variable is numerical, so it can be included as an independent variable in the linear regression model to explain the change of the dependent variable. Moreover, the coded variable can still carry all the information of the original category variable, and the regression equation based on the coded variable also has clear, practical significance. The odds ratio (OR) compares the likelihood that an event will occur in one group to that in a different group based on the occurrence ratio. OR > 1, positively correlated; OR < 1, negatively correlated; OR = 1, not correlated. Variance inflation factors were calculated to quantify the severity of multicollinearity in the multivariate linear regression model. We conducted histogram and Shapiro–Wilk tests to determine the residual distribution, and we found the residuals were a well-modeled normal distribution. The Wilcoxon signed-rank test was performed to compare non-normally distributed data. A *P*-value of less than 0.05 was considered statistically significant. Additionally, stratification analysis was performed based on sex, urban and rural residence, hypertension, and diabetes. All statistical analyses were performed using SPSS 24.0 (IBM Corp., Armonk, NY, USA).

### Role of the funding source

The funder had no role in the study design, data collection, data analysis, data interpretation, or writing of the report. The corresponding author had full access to all the data used in the study and had final responsibility for the decision to submit the manuscript for publication.

## Results

### General characteristics

The analysis included 215,041 individuals (102,692 male and 112,349 female) who completed the questionnaire survey and had a reliable diagnosis of CCVD. Supplementary Material 3–Supplementary Figure 1 shows the data quality control for the SSAPUR study participants. And the distribution of this population by risk factors and general characteristics is shown in Table 1.

**TABLE 1:** The based characteristics of participants with (or without) cardiac-cerebral vascular disease.

Factor	Total (proportion)	Cardiac-cerebral vascular disease		*P*-value
		No	Yes	
Urban and rural				<0.001
Town	111,940 (0.521)	84,069 (0.391)	27,871 (0.13)	
Rural area	103,101 (0.479)	75,059 (0.349)	28,042 (0.13)	
Age (years)				<0.001
60–64	70,913 (0.33)	52,232 (0.243)	18,681 (0.087)	
65–69	50,723 (0.236)	37,934 (0.176)	12,789 (0.059)	
70–74	35,760 (0.166)	26,448 (0.123)	9,312 (0.043)	
75–79	28,131 (0.131)	20,834 (0.097)	7,297 (0.034)	
80–84	18,426 (0.086)	13,586 (0.063)	4,840 (0.023)	
≥85	11,088 (0.052)	8,094 (0.038)	2,994 (0.014)	
Sex				<0.001
Female	112,349 (0.522)	81,370 (0.378)	30,979 (0.144)	
Male	102,692 (0.478)	77,758 (0.362)	24,934 (0.116)	
Education level				<0.001
Have not attended school (including literacy classes)	63,102 (0.294)	43,934 (0.205)	19,168 (0.089)	
Primary school (including private school)	89,059 (0.415)	66,476 (0.31)	22,583 (0.105)	
Junior high school	40,508 (0.189)	31,952 (0.149)	8,556 (0.04)	
High school/secondary school/vocational high school	15,087 (0.07)	11,229 (0.052)	3,858 (0.018)	
College	4,269 (0.02)	3,237 (0.015)	1,032 (0.005)	
Bachelor degree and above	2,322 (0.011)	1,739 (0.008)	583 (0.003)	
Marriage				<0.001
Married	155,973 (0.725)	116,240 (0.541)	39,733 (0.185)	
Widowed	54,142 (0.252)	39,278 (0.183)	14,864 (0.069)	
Divorce	1,795 (0.008)	1,332 (0.006)	463 (0.002)	
Never married	3,131 (0.015)	2,278 (0.011)	853 (0.004)	
Live alone				<0.001
No	186,118 (0.866)	138,148 (0.642)	47,970 (0.223)	
Yes	28,923 (0.134)	20,980 (0.098)	7,943 (0.037)	
Smoking				0.023
No	14,533 (0.068)	10,871 (0.051)	3,662 (0.017)	
Yes	200,508 (0.932)	148,257 (0.689)	52,251 (0.243)	
Drinking				<0.001
Never or occasionally	211,936 (0.986)	156,687 (0.729)	55,249 (0.257)	
1–2 times a week	849 (0.004)	663 (0.003)	186 (0.001)	
At least 3 times a week	1,970 (0.009)	1,556 (0.007)	414 (0.002)	
Often drunk	286 (0.001)	222 (0.001)	64 (0.000)	
Sleep				<0.001
Very good	3,145 (0.015)	2,475 (0.012)	670 (0.003)	
Better	6,531 (0.03)	5,020 (0.023)	1,511 (0.007)	
General	200,588 (0.933)	148,028 (0.688)	52,560 (0.244)	
Relatively poor	3,993 (0.019)	3,020 (0.014)	973 (0.005)	
Very bad	784 (0.004)	585 (0.003)	199 (0.001)	
Workout				<0.001
Never exercise	105,213 (0.489)	76,957 (0.358)	28,256 (0.131)	
Less than once	9,453 (0.044)	6,956 (0.032)	2,497 (0.012)	
Once or twice	27,582 (0.128)	20,499 (0.095)	7,083 (0.033)	
Three to five times	26,339 (0.122)	19,618 (0.091)	6,721 (0.031)	
Six times and above	46,454 (0.216)	35,098 (0.163)	11,356 (0.053)	
Hypertension				0.121
No	135,768 (0.631)	100,619 (0.468)	35,149 (0.163)	
Yes	79,273 (0.369)	58,509 (0.272)	20,764 (0.097)	
Diabetes				0.951
No	196,319 (0.913)	145,270 (0.676)	51,049 (0.237)	
Yes	18,722 (0.087)	13,858 (0.064)	4,864 (0.023)	
Stomach disease				0.173
No	176,690 (0.822)	130,855 (0.609)	45,835 (0.213)	
Yes	38,351 (0.178)	28,273 (0.131)	10,078 (0.047)	
Medical insurance				0.957
No	213,081 (0.991)	157,676 (0.733)	55,405 (0.258)	
Yes	1,960 (0.009)	1,452 (0.007)	508 (0.002)	
Elderly people in your home who require care				0.658
No	189,767 (0.882)	140,396 (0.653)	49,371 (0.23)	
Yes	25,274 (0.118)	18,732 (0.087)	6,542 (0.03)	
Gainful employment				0.555
No	193,528 (0.9)	143,172 (0.666)	50,356 (0.234)	
Yes	21,513 (0.1)	15,956 (0.074)	5,557 (0.026)	
Economic status				<0.001
Very generous	2,738 (0.013)	2,060 (0.01)	678 (0.003)	
Relatively ample	31,721 (0.148)	23,676 (0.11)	8,045 (0.037)	
Basically enough	126,650 (0.589)	94,030 (0.437)	32,620 (0.152)	
Tougher	45,135 (0.21)	32,910 (0.153)	12,225 (0.057)	
Very difficult	8,797 (0.041)	6,452 (0.03)	2,345 (0.011)	
Not participating in public welfare activities				0.007
No	97,774 (0.455)	72,625 (0.338)	25,149 (0.117)	
Yes	117,267 (0.545)	86,503 (0.402)	30,764 (0.143)	
No external abuse				0.569
No	9,623 (0.045)	7,097 (0.033)	2,526 (0.012)	
Yes	205,418 (0.955)	152,031 (0.707)	53,387 (0.248)	
Non-spiritual cultural life				<0.001
No	198,149 (0.921)	146,924 (0.683)	51,225 (0.238)	
Yes	16,892 (0.079)	12,204 (0.057)	4,688 (0.022)	

A total of 55,913 individuals with CCVD were identified among the 215,041 participants. The prevalence distribution and sample size distribution of CCVD in 31 provinces of China were investigated, as shown in Supplementary Material 3–Supplementary Figures 2, 3. A relatively higher proportion and larger sample size of CCVD individuals can be found in eastern China, such as Jiangsu Province, Zhejiang Province, and Shandong Province. The overall prevalence of CCVD among the Chinese elderly was 26%. Meanwhile, there were significant correlations between CCVD and “urban and rural,” “age,” “sex,” “education level,” “marriage,” “live alone” (*P* < 0.001), “smoking” (*P* = 0.023), “drinking” (*P* < 0.001), “sleep” (*P* < 0.001), “workout” (*P* < 0.001), “economic status” (*P* < 0.001), “not participating in public welfare activities” (*P* = 0.007), and “non-spiritual cultural life” (*P* < 0.001) (Table 1).

### Univariate and multivariate logistic regression analysis

Univariate logistic regression analysis examined the associations between each risk factor and CCVD. CCVD (yes or not) was included as the dependent variable, the influencing factors to be considered are as follows: urban and rural residency, age, sex, education level, marriage, living alone, smoking, drinking, sleeping, working out, hypertension, diabetes, stomach disease, medical insurance, whether other older adults in the same home require care, whether engaged in gainful employment, economic status, not participating in public welfare activities, no external abuse, and non-spiritual cultural life (Supplementary Material 3–Supplementary Table 1). The reference group for each variable can also be found in Supplementary Material 3–Supplementary Table 1, and this group is labeled “OR = 1.” Also, the same reference group was used for the variables for subsequent statistical analysis.

**FIGURE 1:**
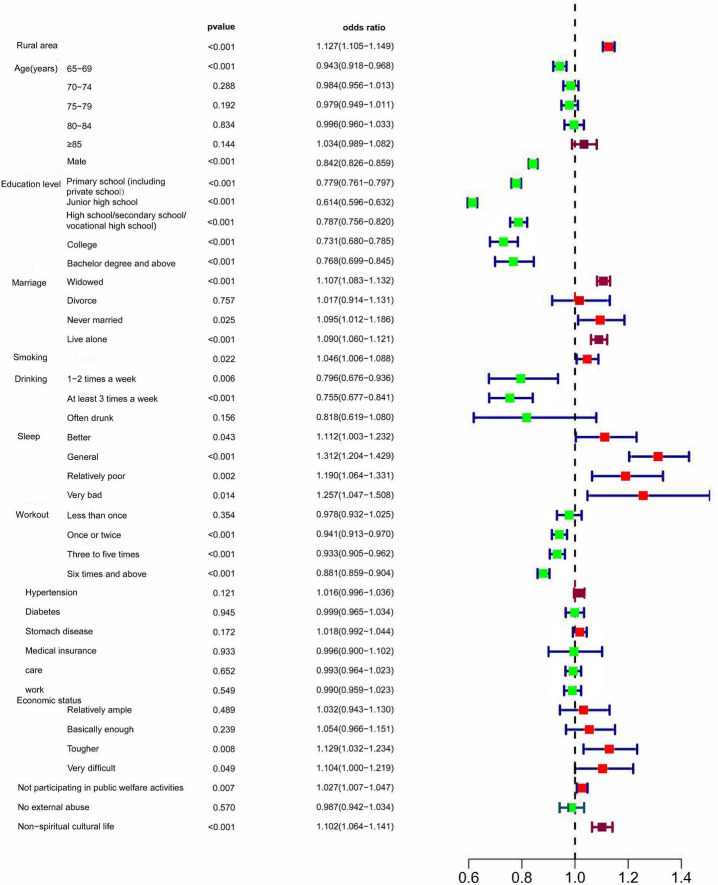
Univariable logistic proportional regression analyses show associations between risk factors and CCVD.

The univariate logistic regression analysis results are presented in Figure 1 and Supplementary Material 3–Supplementary Table 1, which shows the odds ratios (OR) and 95% confidence intervals (95% CI) between clinically relevant factors and CCVD. Rural residency, females, smoking, lower education level, widowed folks, living alone, bad sleep, lack of exercise, poor financial condition, non-participation in public welfare activities, and non-spiritual cultural life were risk variables for CCVD that were statistically significant. (1) Rural residents are at higher risk than urban residents (OR = 1.127, 95% CI: 1.105–1.149, *P* < 0.001). (2) Compared with females, males have a lower risk of CCVD (OR = 0.842, 95% CI: 0.826–0.859, *P* < 0.001). (3) Smoking is a risk factor for CCVD. The difference is statistically significant (OR = 1.046, 95% CI: 1.006–1.088, *P* < 0.05). (4) In terms of education, the higher the education level, the lower the prevalence of CCVD (*P* < 0.001). (5) Widowed individuals have a higher prevalence of CCVD than those who are not widowed (OR = 1.107, 95% CI 1.083–1.132, *P* < 0.001). (6) “Lives alone” is also a significant risk factor for CCVD (OR = 1.090, 95% CI: 1.060–1.121, *P* < 0.001). (7) The worse the sleep status, the higher the prevalence of CCVD (*P* < 0.001). (8) Regular exercise is a protective factor for CCVD (*P* < 0.001). (9) Economic status is a predictive index for CCVD, with worse financial situations linked to the occurrence of CCVD (*P* < 0.01). (10) “Not participating in public welfare activities” (OR = 1.027, 95% CI: 1.007–1.047, *P* = 0.007) and (11) “non-spiritual cultural life” (OR = 1.102, 95% CI: 1.064–1.141, *P* < 0.001) are significant risk factors for CCVD. Interestingly, we divided the population into six groups by age: 60–64, 65–69, 70–74, 75–79, 80–84, and ≥85 groups, and the results show that the difference is statistically significant (*P* < 0.001), but this only occur in the “65–69” group (OR = 0.943, 95% CI: 0.918–0.968, *P* < 0.001). It shows that in the elderly population (≥60 years old), age does not increase or even decrease the risk of CCVD. In addition, moderate drinking is a protective factor for CCVD. The difference was statistically significant (*P* < 0.001).

**FIGURE 2:**
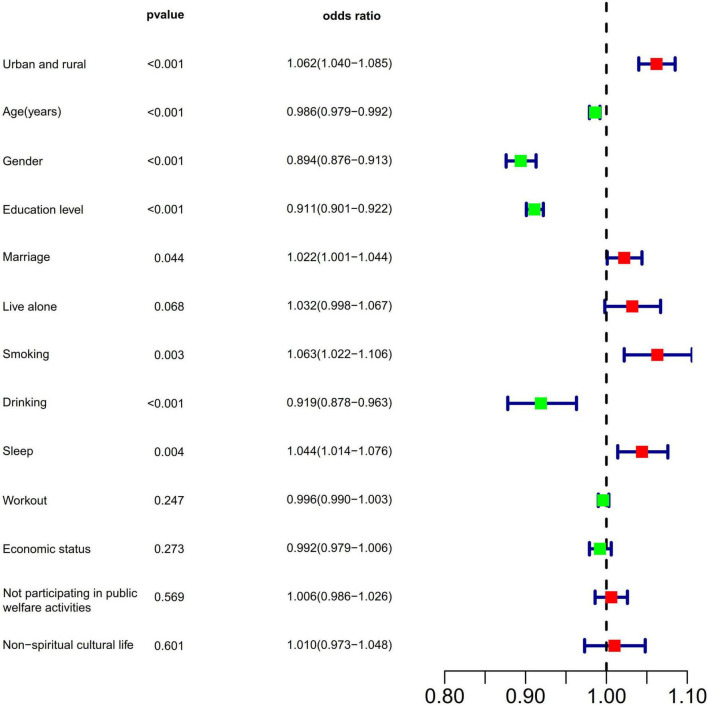
Multivariable logistic proportional regression analyses show associations between risk factors and CCVD.

Multivariate logistic regression was used to exclude other confounding factors’ influence and determine the association between predictors and CCVD. Those test results reaching a liberal statistical threshold of *P* < 0.05 were then entered into a multivariable logistic regression analysis. Significant factors for CCVD are as follows: “Urban and rural” (OR = 1.062, 95% CI: 1.040–1.085, *P* < 0.001), “age” (OR = 0.986, 95% CI: 0.979–0.992, *P* < 0.001), “sex” (OR = 0.894, 95% CI: 0.876–0.913, *P* < 0.001), “education level”(OR = 0.911, 95% CI: 0.901–0.922, *P* < 0.001), “marriage”(OR = 1.022, 95% CI: 1.001–1.044, *P* < 0.05), “smoking” (OR = 1.063, 95% CI: 1.022–1.106, *P* = 0.003), “drinking” (OR = 0.919, 95% CI: 0.878–0.963, *P* < 0.001), and “sleep” (OR = 1.044, 95% CI: 1.014–1.076, *P* = 0.004) (Figure 2, Supplementary Material 3–Supplementary Table 1). The results show that female rural residents, low literacy levels, smoking, bereavement, and poor sleep quality are risk factors for prevalent CCVD. And moderate alcohol consumption can reduce CCVD risk. Especially, increasing age does not increase or even decrease the risk of CCVD.

To ensure that the significant risk factors genuinely impacted CCVD, we further analysis of CCVD and its associated risk factors. In the multivariate linear regression model, holding all other variables at any fixed value, CCVD remained associated with “urban and rural” (β = 0.012, *P* < 0.001), “age” (β = −0.003, *P* < 0.001), “sex” (β = −0.022, *P* < 0.001), “education level” (β = −0.017, *P* < 0.001), “marriage” (β = 0.004, *P* = 0.047), “smoking” (β = 0.012, *P* = 0.003), “drinking” (β = −0.015, *P* = 0.001), and “sleep” (β = 0.008, *P* = 0.005). Additionally, we made a collinearity diagnosis and considered variable collinearity during the model fitting phase. The results show that there is no collinearity problem between these factors (Table 2).

**TABLE 2:** Correlative factors’ effect on cardiac-cerebral vascular disease based on multiple linear regression analysis.

Factors	Cardiac-cerebral vascular disease		
	β	*P*-value	VIF
Urban and rural	0.012	<0.001	1.212
Age	−0.003	<0.001	1.153
Sex	−0.022	<0.001	1.142
Education level	−0.017	<0.001	1.381
Marriage	0.004	0.047	1.542
Live alone	0.006	0.065	1.435
Smoking	0.012	0.003	1.054
Drinking	−0.015	0.001	1.055
Sleep	0.008	0.005	1.080
Workout	−0.001	0.247	1.235
Economic status	−0.002	0.257	1.121
Not participating in public welfare activities	0.001	0.550	1.069
Non-spiritual cultural life	0.002	0.542	1.108

β: parameter estimate.

### Stratification analysis

To control for confounding factors, stratification analysis was performed based on sex, urban and rural residence, hypertension, and diabetes to estimate the relationship between parameters and CCVD within each layer.

**FIGURE 3:**
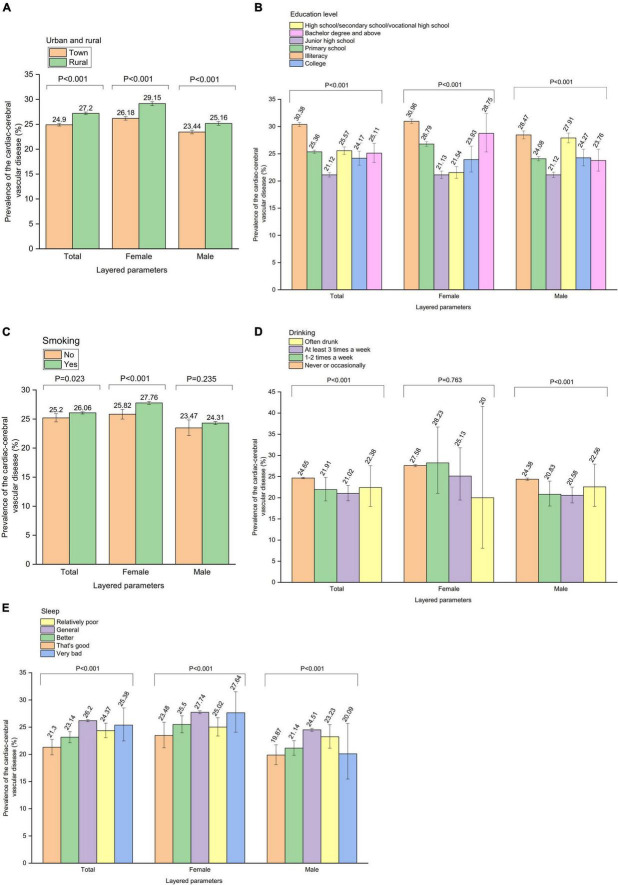
The analysis of the relationship between parameters and cardiac-cerebral vascular disease was made further by gender stratification. Prevalence of the cardiac-cerebral vascular disease by **(A)** “Urban and rural” stratification, **(B)** “Education level” stratification, **(C)** “Smoking” stratification, **(D)** “Drinking” stratification, **(E)** “Sleep” stratification.

The relationship between parameters and CCVD was further analyzed by sex stratification. A total of 30,979 female individuals with CCVD were identified among 112,349 female participants, and the overall prevalence of CCVD among elderly Chinese females was 27.6%. In terms of females, there were significant correlations between CCVD and “urban and rural,” “education level,” “marriage,” “lives alone,” “smoking,” “sleep,” “workout,” “economic status,” and “non-spiritual cultural life” (*P* < 0.001). In addition, 24,934 male individuals with CCVD were identified among 102,692 male participants, and the overall prevalence of CCVD among elderly Chinese males was 24.3%. In terms of males, there were significant correlations between CCVD and “urban and rural” (*P* < 0.001), “age” (*P* < 0.001), “education level” (*P* < 0.001), “marriage” (*P* < 0.001), “lives alone” (*P* = 0.004), “drinking” (*P* < 0.001), “sleep” (*P* < 0.001), “workout” (*P* = 0.012), “economic status” (*P* = 0.010), and “non-spiritual cultural life” (*P* = 0.022) (Figure 3, Supplementary Material 3–Supplementary Table 2).

**FIGURE 4:**
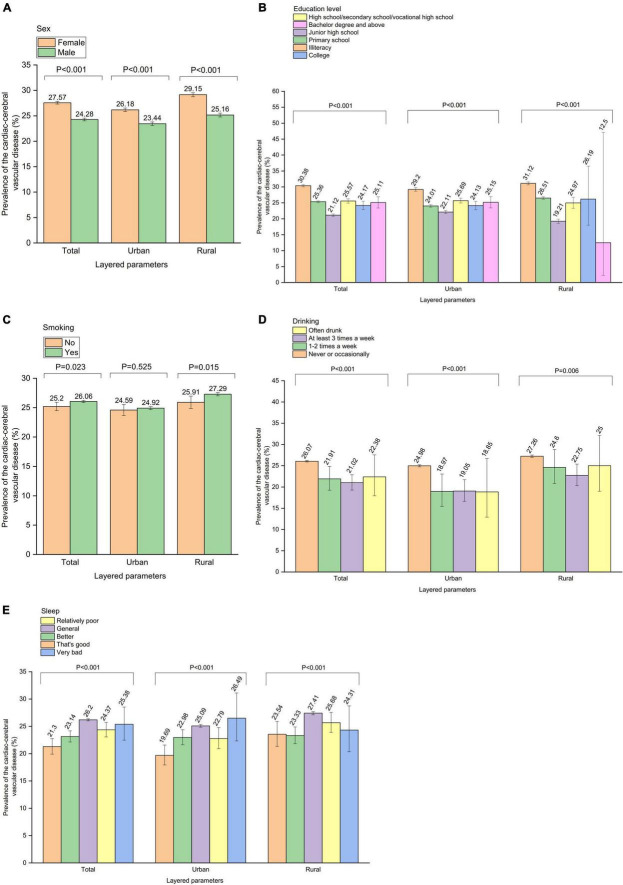
The relationship between parameters and cardiac-cerebral vascular disease was further analyzed by “Urban and rural” stratification. Prevalence of the cardiac-cerebral vascular disease by **(A)** “Sex” stratification, **(B)** “Education level” stratification, **(C)** “Smoking” stratification, **(D)** “Drinking” stratification, **(E)** “Sleep” stratification.

The relationship between parameters and CCVD was further analyzed by “urban and rural” stratification. A total of 27,871 individuals with CCVD were identified among 111,940 urban participants, and the overall prevalence of CCVD among the urban Chinese elderly was 24.9%. In terms of urban residence, there were significant correlations between CCVD and “age” (*P* < 0.001), “sex” (*P* < 0.001), “education level” (*P* < 0.001), “marriage” (*P* < 0.001), “lives alone” (*P* = 0.005), “drinking” (*P* < 0.001), “sleep” (*P* < 0.001), “workout” (*P* = 0.002), “economic status” (*P* < 0.001), “not participating in public welfare activities” (*P* = 0.045), and “non-spiritual cultural life” (*P* = 0.004). In addition, a total of 28,042 individuals with CCVD were identified among 103,101 rural participants, and the overall prevalence of CCVD in the rural Chinese elderly was 27.2%. In terms of rural residence, there were significant correlations between CCVD and “age” (*P* < 0.001), “sex” (*P* < 0.001), “education level” (*P* < 0.001), “marriage” (*P* < 0.001), “lives alone” (*P* < 0.001), “smoking” (*P* = 0.015), “drinking” (*P* = 0.006), “sleep” (*P* < 0.001), “workout” (*P* < 0.001), “hypertension” (*P* = 0.004), “not participating in public welfare activities” (*P* = 0.008), and “non-spiritual cultural life” (*P* = 0.002) (Figure 4, Supplementary Material 3–Supplementary Table 3).

**FIGURE 5:**
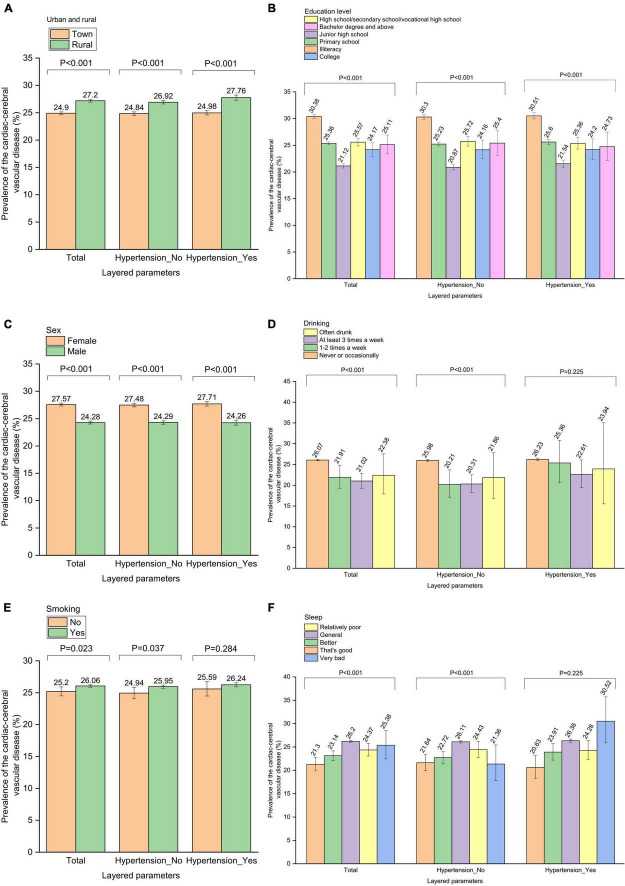
The relationship between parameters and cardiac-cerebral vascular disease was further analyzed by “hypertension” stratification. Prevalence of the cardiac-cerebral vascular disease by **(A)** “Urban and rural” stratification, **(B)** “Education level” stratification, **(C)** “Sex” stratification, **(D)** “Drinking” stratification, **(E)** “Smoking” stratification, **(F)** “Sleep” stratification.

The relationship between parameters and CCVD was further by analyzing hypertension stratification. Among the 135,768 participants without hypertension, 35,149 people were found to have CCVD, making the overall prevalence of the condition among Chinese seniors without hypertension 25.9%. Out of 79,273 participants with hypertension, 20,764 people had CCVD, making it the 26.2% prevalence of CCVD overall among Chinese elderly people with hypertension (Figure 5, Supplementary Material 3–Supplementary Table 4).

**FIGURE 6:**
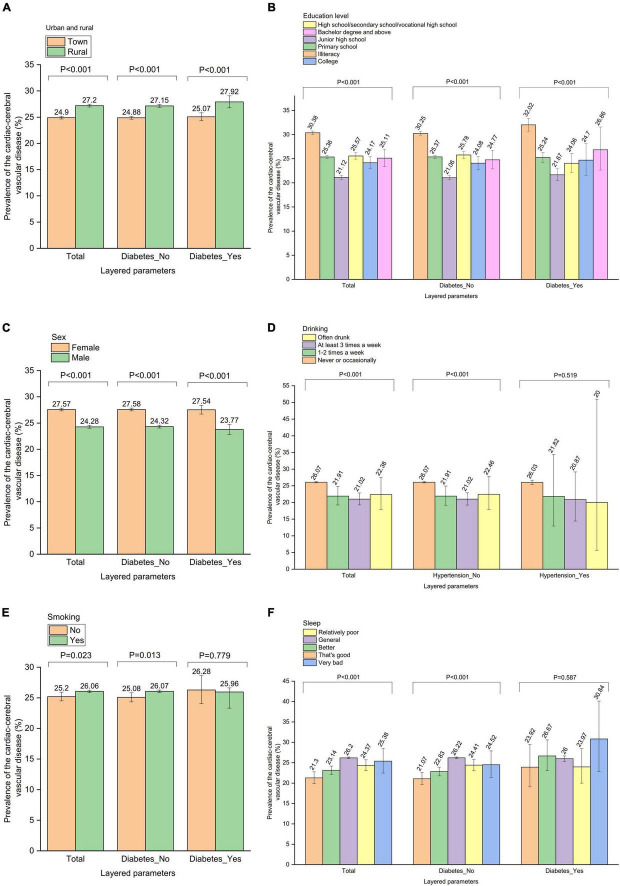
The relationship between parameters and cardiac-cerebral vascular disease was further analyzed by “diabetes” stratification. Prevalence of the cardiac-cerebral vascular disease by **(A)** “Urban and rural” stratification, **(B)** “Education level” stratification, **(C)** “Sex” stratification, **(D)** “Drinking” stratification, **(E)** “Smoking” stratification, **(F)** “Sleep” stratification.

The relationship between parameters and CCVD was further analyzed by diabetes stratification. Among 196,319 participants without diabetes, 51,049 people were found to have CCVD, making the overall prevalence of the condition in Chinese seniors without diabetes 26.0%. Eighteen thousand seven hundred twenty-two participants with diabetes had a total of 4,864 cases of CCVD, making the prevalence of CCVD in old Chinese diabetics overall 26.0% (Figure 6, Supplementary Material 3–Supplementary Table 5).

## Discussion

Our findings show that significant risk factors for CCVD among the Chinese elderly include rural residence, being female, having a low literacy level, smoking, alcohol abuse, and poor sleep quality. CCVD is thus a significant health problem among the elderly in China. The study showed that the incidence of CCVD increased with age, and the number of patients >60 years old increased significantly compared with other age groups. The prevalence of CCVD was significantly higher in men than women, and the probability of developing CCVD in people >60 years old was significantly higher than in other age groups. The prevalence of stroke, coronary heart disease, and lacunar infarction is also relatively high in this group. In addition, mortality in patients with CCVD increases significantly with age (11, 12). Currently, CCVD prevalence and mortality in China are still on the rise, with prevalence directly proportional to age.

Multivariate analysis showed that the prevalence of CCVD in the elderly is significantly related to whether individuals live in urban or rural areas, sex, education level, smoking, drinking, and poor sleep quality. Our study suggests that living in a rural area is a significant risk factor for CCVD in the elderly. This survey showed that being female is a significant risk factor for CCVD in the elderly. Being female and elderly are independent risk factors and may be related to women’s physiological characteristics, education level, socioeconomic background, family status, and many other factors that are different from those of men (13, 14). Among married women, marital stress had a significant impact on the occurrence of CCVD (OR = 2.9, 95% CI: 1.3–6.0). After adjusting for age, estrogen level, education level, smoking, drinking, triglycerides, high-density lipoprotein cholesterol level, and other factors, the risk of female central cerebrovascular disease is still increasing (13). In addition, women with acute myocardial infarction are more likely to experience increased psychological distress than men (14). Similarly, compared with non-smokers, women who smoke have a 25% higher relative risk of CCVD than men (15, 16). The mortality rate of CCVD in men in all age groups in China is significantly higher than that in women. However, in the elderly population, the risk of CCVD in women is significantly higher, and the proportion of deaths from cardiovascular diseases among total deaths in the female population exceeds that of men (17).

This survey showed that the lower the level of education, the greater the risk of CCVD in the elderly. A large study that analyzed nearly 90,000 people in Australia and New Zealand found that compared with people with a higher level of education, people with primary education had an increased prevalence and risk of all-cause death (18). The correlation between lower education levels and increased prevalence of CCVD may also be related to behavioral risk factors, such as smoking, obesity, and lack of exercise (19, 20). The prevalence of central cerebrovascular disease in the population with a lower education level is significantly higher than that of patients with middle and high levels of education. The univariate analysis in this study revealed a negative correlation between educational attainment and the total prevalence of cardiovascular and cerebrovascular disease (*r* = −0.45; *P* < 0.001). Education level is independently related to the risk of CCVD. It has been shown that education level can be considered the best predictor of global cardiovascular risk for hypertensive patients and should be evaluated in hypertension and cardiovascular risk management strategies (21). Data from another study that evaluated the impact of education level on CCVD also showed a strong association between a low level of education and heart disease (22).

We found that smoking was a significant risk factor for cardiovascular and cerebrovascular diseases in the elderly. Smoking increased all-cause mortality. A study that observed the dose-response relationship between smoking and death found that, compared with people who never smoked, the mortality rate in former smokers decreased significantly with the extension of smoking cessation time (23). Smoking among the elderly is a significant risk factor for CCVD (24). A 2015 meta-analysis studied people aged 60 years and older and found that smoking was closely related to acute coronary events, strokes, and cardiovascular deaths. Compared with people who have never smoked, the risk of death due to CCVD in smokers doubles and increases with increasing smoking dose; however, the threat continues to decrease with the extension of smoking cessation time. Smoking is a strong independent risk factor for cardiovascular and cerebrovascular events and mortality even in old age. It increases the mortality of CCVD by more than five times, suggesting that smoking cessation in the elderly remains beneficial in reducing their risk (25).

We found moderate drinking was a protective factor for CCVD in the elderly. Studies have shown a consistent J-shaped association between alcohol consumption and various adverse health consequences, including coronary heart disease, diabetes, hypertension, congestive heart failure, stroke, dementia, and all-cause death. Light to moderate drinking (up to one drink per day for women and one to two drinks per day for men) provides benefits related to heart protection, while excessive drinking can cause heart health problems (26). Another study also showed that habitual light to moderate drinking is associated with a lower risk of overall mortality, coronary heart disease, diabetes, congestive heart failure, and stroke. However, excessive drinking is associated with an increased risk of CCVD (27). Excessive drinking may increase the risk of death and CCVD (28).

This survey found that poor sleep quality is a significant risk factor for CCVD in the elderly. Whether due to sleep disturbances or lack of proper sleep patterns, it is related to the occurrence and development of CCVD (29). Studies have shown that short sleep time is associated with an increased risk of developing CCVD or death (30, 31). Another study involving 25,025 participants found that sleep time has a U-shaped relationship with the mortality of cardiovascular and cerebrovascular diseases; this study also found that sleep time is an independent risk factor for death from CCVD in women. A sleep time of less than 5 h or more than 10 h is linked with the greatest risk of death from cardiovascular and cerebrovascular diseases (32). Severe sleep disturbance has been shown to increase the risk of first CCVD (OR = 1.80, 95% CI: 1.07–3.03) and coronary heart disease events (OR = 1.97, 95% CI: 1.09–3.56) (33).

We found that bereavement and being unmarried were significant risk factors for CCVD in the elderly. In contrast, the results of another study showed that compared with married stroke patients, the mortality rate of unmarried (OR = 0.69), divorced (OR = 0.69), and widowed (OR = 0.80) patients was lower. The stroke fatality rate of unmarried, divorced, and widowed patients was lower than that of married stroke patients (34).

Our survey results suggest that living alone is a significant risk factor for CCVD in the elderly. Other studies have shown that the morbidity and mortality of CCVD are higher among older people living alone. This results from their isolation or the stress of going through a period of sorrow (35). Older people with little social contact are twice as likely to die than those who do not live alone. The following logic can usually explain this fact. Social relations promote physical activity, improve mental state, and provide assistance during illness, all of which help an individual to restore their health (36).

This survey found that regular exercise is a significant protective factor for CCVD in the elderly. Following long-term resistance training, healthy older people show strong muscle oxygen uptake capacity, vascular regulation, and vascular endothelial function. According to some reports, endurance training can improve the matching of microvascular blood flow, oxygen supply and utilization, muscle oxidation capacity, and oxygen saturation. Walking exercise can enable the elderly with peripheral artery disease to obtain better muscle oxygen supply and uptake, which positively affects lung ventilation and oxygenation (37, 38). A longitudinal cohort study showed that communities with basic sports facilities could increase the activities of elderly individuals and reduce their risk of cardiovascular and cerebrovascular diseases (39).

We found that low economic status was a significant risk factor for CCVD in the elderly. For every US$10,000 increase in income, the death rate associated with CCVD was reduced by 10%. Consistent with this, an American study found that an increase in the prevalence of CCVD was related to low-income individuals and communities. Individuals with poor financial status often engage in risky behaviors such as drinking alcohol and smoking. These socioeconomic variables influence behavioral variables synergistically and can be utilized to estimate the risk of cardiovascular and cerebral disorders (40). In addition, residents in low-income areas may receive fewer physical examinations each year and, at the same time, cannot afford medical expenses for CCVD treatment (41).

Finally, the results of this survey found that “never participating in public welfare activities” and “life without spiritual culture” were significant risk factors for CCVD in the elderly.

There are some limitations to our research. First, we did not make a detailed distinction between various manifestations of CCVD. For example, we did not separately investigate the incidence of coronary heart disease, stroke, pulmonary heart disease, heart failure, rheumatic heart disease, and congenital heart disease among the elderly in China. Second, similar to other large-scale population surveys, the diagnosis of CCVD was primarily based on standardized questionnaires. This could result in misdiagnosing other non-vascular brain diseases (such as neuralgia, intracranial infection, intracranial space-occupying lesions, extracranial head and face diseases, etc.), since all of these conditions can cause headaches, particularly in the elderly. Another limitation of a diagnosis based on the questionnaire is that we may miss those patients with congenital heart disease, a subtype of CCVD. A small number of congenital heart diseases can heal themselves before the age of 5 years, and a small number of patients have mild deformities that have no evident impact on circulatory function, and thus they are not diagnosed. Misdiagnosis of other non-vascular brain diseases may lead to overestimating the prevalence of CCVD. Conversely, we may have underestimated the prevalence of CCVD by ignoring the subtypes of congenital heart disease, especially in areas where medical resources are scarce. Finally, our data related to drinking only included qualitative concepts, so we could not assess the impact of specific quantities of alcohol on CCVD.

## Conclusion

In conclusion, this comprehensive national study demonstrates a high prevalence of CCVD and that it is a chronic disease that is not well treated or controlled among the elderly in China. Major risk factors for prevalent CCVD among the elderly in China include the following: rural residence, being female, low literacy level, poor sleep quality, bereavement, non-marriage, living alone, lack of exercise, poor financial situation, and non-participation in public welfare activities.

## Data availability statement

The original contributions presented in this study are included in the article/Supplementary material, further inquiries can be directed to the corresponding author.

## Ethics statement

The research protocol was approved by the Ethical Review Committee of Beijing Hospital (2021BJYYEC-294-01) and the National Bureau of Statistics (No. [2014] 87). All participants provided written informed consent to participate in this study. Written informed consent was obtained from the individual(s) for the publication of any potentially identifiable images or data included in this article.

## Author contributions

LM and JX participated in the topic selection, design of the manuscript, involved in data analysis and interpretation, and subsequent revision of the manuscript. JiL, JH, HX, DW, and XH participated in data analysis and interpretation. XZ, QZ, JuL, TG, and DL drafted and revised key theories in the manuscript and answered the academic questions. All authors agreed to be published.
