# Supplementary material for: Self-reported prevalence and potential factors influencing cardio-cerebral vascular disease among the Chinese elderly: A national cross-sectional study
Source: Front Cardiovasc Med. 2022 Oct 20;9:979015. doi: 10.3389/fcvm.2022.979015 (PMC9630358; doi:10.3389/fcvm.2022.979015)
Supplement: Supplementary file 1 [file Data_Sheet_1.docx]

**Supplementary Appendix**

**This appendix has been provided by the authors to give readers additional information about their work**

**Supplement to: Prevalence, risk factors, and management of cardiac-cerebral vascular disease in the China elderly: a national cross-sectional study**

**CONTENTS**

**Supplement 1.** Sampling plan of the 4th Survey of the Aged Population in Urban and Rural China (SSAPUR)

**Supplement 2.** Personal questionnaire of the 4th SSAPUR

**Supplement 3.** Supplemental figures and tables of results

**Supplement 3-figure S1.** Flowchart of data quality control for the 4th Survey of the Aged Population in Urban and Rural China (SSAPUR) study participants.

**Supplement 3-figure S2.** Geographic distribution of the prevalence of CCVD in 31 selected provinces/municipalities in the 4th Survey of the Aged Population in Urban and Rural China (SSAPUR) study. The numbers in parentheses indicate the prevalence of CCVD in each location.

**Supplement 3-figure S3.** Geographic distribution of the prevalence of CCVD in 31 selected provinces/municipalities in the 4th Survey of the Aged Population in Urban and Rural China (SSAPUR) study Numbers in parentheses indicate the sample size from each location.

**Supplement 3-table S1**. Univariate and multivariate logistic analysis for cardiac-cerebral vascular disease.

**Supplement 3-table S2**. The analysis of the relationship between parameters and cardiac-cerebral vascular disease by gender stratification

**Supplement 3-table S3**. The analysis of the relationship between parameters and cardiac-cerebral vascular disease by “urban and rural” stratification

**Supplement 3-table S4**. The analysis of the relationship between parameters and cardiac-cerebral vascular disease by hypertension stratification

**Supplement 3-table S5**. The analysis of the relationship between parameters and cardiac-cerebral vascular disease by diabetes stratification

**Supplement 1. Sampling plan of the 4th Survey of the Aged Population in Urban and Rural China (SSAPUR)**

**Total sample size setting**

The adequate sample size is the basic premise to ensure survey representativeness and data accuracy. In this survey, a total of 221,760 senior citizens were selected as the total sample survey object. The sampling technique demonstration process is as follows.

Usually, in the data analysis of a sample survey, proportion is the main objective of the analysis. Therefore, the proportion estimation method is used to estimate the total sample size of the survey. In the fourth survey, the maximum variance of the core statistical index "proportion of healthy elderly people" is planned to be selected, that is, when P=0.5, under the condition of confidence of 95.45% (probability degree t=2), the absolute sampling error d is not more than 2%, and the sample size N0 =t2Xp(1-p)/d2=2500.

Because of the stratified multi-stage complex sampling, the design effect would normally be between 2 and 3, In this scheme, the design effect DEFF is set as 2.8, so that the initial size of the total sample size is 2.8x2500 =7000. Considering the analysis of future data by urban and rural, provincial, gender, and age groups (usually divided into 7 age groups of 60-64, 65-69, 70-74, 75-79, 80-84, 85-89, and 90 years old and above), the total sample size should continue to increase (using the "flexible method") =29.46184 times:29. 46184x 7000=206233. Considering that during the implementation of the survey, there may be some cases of household separation, population movement, death, lack of access, lack of qualified respondents' ability to answer, and other non-answers caused by various reasons, assuming that the cumulative amount accounts for 7%, then we enlarge the total number of samples to 206,233 //0.93= 221,756, and the multiple of 20 is 221,760. It is estimated that by the end of August 2015, the size of the elderly population in China will be about 221 million, so the total sampling ratio of this survey is about 1%.

**Survey sampling box**

Based on the administrative division used by the Sixth General Administration in 2010, and combined with the change data of the administrative division in 2014 recently released by the Ministry of Civil Affairs, a five-level sampling frame of "one province, one district (county)-- sub-district (town)-- one residential (village) committee -- the elderly" was compiled.

**Determination of sampling unit and extraction method**

The fourth survey will cover the whole country (including 22 provinces, 5 autonomous regions, and 4 municipalities directly under the central government; Excluding Taiwan, Hong Kong and Macao), a total of 2,85districtsct (county) administrative units are selected as primary sampling units, from which a certain number the of district (county) are selected as primary sampling units (PSU).

**The simple sampling idea is as follows:**

1, Determine the sampling ratio, 1/1000

2. The sample number shall be allocated according to the proportion of the elderly population in each province in the whole country

3. Determine the sampling number of counties, towns, and communities, and select 462 counties. According to PPS sampling, select 4 towns from each county and 4 communities (village or neighborhood committee) from each township.

4. Select 30 elderly people from each community

**Specific sampling steps**

According to the theory of stratified multi-stage PPS sampling design, the number of sampling units (PSU) in the first stage has a decisive effect on the total sampling error, while the cumulative error in other stages is relatively small. Therefore, under the condition of controlling the total size of design samples, the number of sampling units in the first stage should be enlarged as far as possible. However, too many districts (counties) will increase the cost and difficulty of the survey. Based on the experience of previous large-scale surveys, 2 investigators were selected from each community and each investigator undertook 15 questionnaires in a month, which was a relatively moderate and efficient workload. Therefore, according to the total sample size of 221,760 designed above, the fourth survey adopts the conservative "reverse order, 30 * 4 * 4 * 462" sample unit allocation method to extract samples step by step:

(1) The number of extraction areas (counties) was determined to be 462 in advance. Firstly, the proportion of the total number of elderly people in the total number of elderly people (aged 65 and above) in each province in the data of the sixth census in 2010 was calculated. Then multiply by 462 (proportional distribution). In the process of operation, considering that the number of provinces (counties) should be an integer, the total number of districts (counties) extracted should be 466 after rounding.

(2) It is determined in advance that the number of streets/towns to be extracted in the district (county) is 4. According to the PPS sampling method, the total number of the elderly population in each district (county) (the number of elderly people aged 65 and above) is allocated within each district (county), rounded to the whole number, and the total number of streets/towns selected should be equal to 4.

(3) The number of residential (village) committees to be selected from the selected streets/towns is determined to be 4 in advance. The total number of elderly people in each street/town (the number of elderly people aged 65 and above) is used as auxiliary information. According to the PS sampling method, it is distributed in each street/town. The number of residential (village) committees extracted should be equal to 4 in total.

(4) In the selected residential (village) committees, according to the list of the elderly reported before the survey, take isometric sampling to select 30 elderly people.

**Supplement 2.** **Personal questionnaire of the 4th SSAPUR**

Printing serial number:

Questionnaire No:

| Article 7 of the Statistics Law of the People's Republic of China stipulates: Organs of state, enterprises, institutions, other organizations, individual businesses, and individuals under statistical investigation, must be following this law and relevant regulations of the state to provide true, accurate, complete and timely data that are necessary for statistical investigation, and shall not provide false or incomplete statistical data nor delay or refuse to provide statistical data.  Article 25 of the Statistical Law of the People's Republic of China stipulates: No unit or individual can provide or divulge the data obtained during statistical investigations that can identify or deduce the identity of any individual statistical objects or use the data for purposes other than statistics. |
| --- |

Table no: CRCA2015-1

Formulating authority: Ministry of Civil Affairs

Approving authority: National Bureau of Statistics

Approval Number: National Control [2014] No. 87

Valid until: September 2015

The fourth sampling survey on the living conditions of the elderly in urban and rural China

Personal questionnaire (long form)

*********************************************************************

I. Interview location:

Provinces (autonomous regions, municipalities directly under the Central Government) _____________________________________________________________________

Prefecture level city________________________________________________________

County (city, district) ___________________________________________________

Township/Sub-district ___________________________________________________

Village (neighborhood) committee _________________________________________

Home address _________________________________________________________

II. Interview Records:

| Interview date | | Start time | End time |
| --- | --- | --- | --- |
| Month | Day |  |  |
|  |  |  |  |
|  |  |  |  |
|  |  |  |  |

Interviewer's signature: _________________ Telephone: _________ Date: __ Month ___Day

Signature of township supervisor: _________ Telephone: _________ Date: __ Month ___ Day

Signature of county supervisor: ___________ Telephone: _________ Date: __ Month ___Day

Signature of input personnel: _____________ Telephone: _________ Date: __ Month ___Day

【 Instructions 】

Dear __________ (name of the interviewee):

I am a staff member of the fourth survey of the living conditions of the elderly. Doing this survey is an important event in the life of the elderly in the nation, and the survey has been approved by the National Working Committee on Aging and the National Bureau of Statistics. We hope that through this survey, we will have a comprehensive understanding of the various situations, difficulties, and problems happened in older adults’ life in the nation, in order to provide a basis for the Party and the government to formulate policies, with the aim of improving the quality and level of life of the elderly nationwide. Luckily, you are selected to be one of the representatives. We will ensure your confidentiality by strictly abiding by the relevant provisions of the Statistics Law. We hope you can tell us the actual situation of your life. Thank you very much for your cooperation!

[Fill in the form]

| 1. The questionnaire must be filled out by the interviewers at interviewees’ homes. All the data must be fair, objective and accurate. 2. There are no right or wrong answers to questions, but the responses must accurately reflect the real situation of the interviewees. 3. There are two types of questions in this questionnaire: one is multiple choice questions, and the other is fill-in-blank questions. 4. Single-choice questions: Please mark one of the answers.   5. Multiple-choice questions: Please mark the box(es) in front of the corresponding choice(s).  6. Fill in the blanks: Please fill in the answers (words or numbers) that reflect the true situation of the interviewee.  7. If you select "Other" that need to specify in detail, please give corresponding explanation.  8. If some questions are not applicable, or older adults cannot answer or refuse to answer, please mark "×" on the number of questions and note the reason next to the questions. |
| --- |

1. Basic situation

A1 Gender of interviewee: [Based on interviewer’s observation]: 1 male 0 female

A2 Date of birth: [Interviewer fills according to the interviewee’s ID card] _____ Year _____ Month

A3 What kind of domicile do you have? [Interviewer fills according to the household registration book]

1. Agricultural 2. Non-agricultural 3. Unified household registration

A4 What ethnicity do you belong to? [Interviewer fills according to the interviewee’s ID card]

1 The Han ethnicity 2 The Zhuang ethnicity 3 The Hui ethnicity

4 The Man ethnicity 5 The Uygur ethnicity 6 The Miao ethnicity 7 The Yi ethnicity 8 The Tujia ethnicity 9 The Tibetan ethnicity

10 The Mongolian ethnicity 11 other ethnicity __________________ (please write the full name of the ethnicity)

A5 Your education level:

1 never attended school (including literacy classes) 2 Primary school (including private schools) 3 Junior high school 4 High school/technical secondary school/vocational high school 5 Junior college 6 Bachelor degree or above

A6 Your professional and technical title: 1 None 2 Technician Level 3 Junior Title 4 Intermediate Title 5 Senior Title

A7 Your political status: 1 general public 2 members of the Communist Party of China

3 democratic parties 4 personages without party affiliation

A8 Your current marital status:

1. Has a spouse whose age is ________?
2. Widowed, widowed ________ years ago?

3 Divorce, divorced ________ years ago?

4 Never married

B． Family situation

[Interviewer: Next, we want to know about your family.]

B1 Status of your current children: [Interviewer: including foster/stepchildren, excluding daughter-in-law/son-in-law]

B1.1 Son _____ (The number of person);

B1.2 daughter _____ (The number of person).

[If the respondent has no children, please go to question B2 directly]

B1.3 Do any of your children have difficulties in life?

1 Yes 0 No [Go question B1.5 directly]]

B1.4 Do you support your child financially on a long-term basis?

1 Yes 0 No

B1.5 Do your children take turns to care for you?

1 Yes 0 No

B1.6 Do you think your children are filial?

1 filial 2 so-so 3 not filial

B2 Who are in your family (living with you) now? (Multiple choices)

[Interviewer: select according to the relationship with the interviewee, and indicate the number of people]

□ Living alone □ Spouse □ The Parents -in-law/Parents _____ (The number of person) □ Son _____ (The number of person) □ daughter-in-law _____ (The number of person) □ daughter _____(The number of person)□ son-in-law_____ (The number of person)□ (Great) grandchildren _____(The number of person)□ personal care assistant_____ (The number of person) □ other people_____(The number of person)

B3 Do you have children residing outside this province?

1 Yes 0 No [Go to question B4 directly]

B3.1 How many of your children are living outside this province? __________ (The number of person)

B3.2 How many times do your children, who live outside the province, visit you each year?

1 less than once 2 once 3 two to three times 4 four times or more

B 4 Are you willing to have a long-term living arrangement with your children?

1 Yes 2 No 3 Depends

B5 Do you now help your children with any of the following things? (Multiple choice) [Interviewer: ask item by item]

□ Take care of the home □ Do housework

*(rural) □ Do farm work □ Take care of grandchildren

□ Others (please specify) _________□ None have been done

B6 How many relatives/friends do you keep in touch with regularly?

B7 What major events have happened to your family this year? (Multiple choice) [Interviewer: ask item by item]

□ Children got unemployed □ Children got divorced

□ Disputes/Lawsuits □ Relatives got a serious illness

□ Loved ones passed away □ Others (please specify) _____

□ None

B8 What kind of transportation do you usually use? (Multiple choice) [Interviewer: Ask item by item]

□ By bike □ By motorcycle/electric bike

□ By bus □ By subway

□ By taxi □ By personal vehicle

□ Others (please specify) __________ □ None

B9 Who is in charge of making major expenses in your family?

1 self 2 spouse 3 children 4 negotiate together

C. Health and medical conditions

[Interviewer: Next, we'd like to know about your medical condition]

C-1 Do you smoke?

1. Never [Go to question C2 directly]

2. I used to smoke, but now I have given up smoking

3 Often

4 Occasionally

C1.1 How many years have you been a smoker? _____

2 Do you drink alcohol?

1. not at all or occasionally 2 .1-2 times a week

3. at least 3 times a week 4 often drunk

C-3 How is your sleep quality?

1. very good 2 relatively good 3 average

4 relatively poor 5 very poor

C-4 Can you see clearly (including wearing glasses)?

1 very clear 2 relatively clear 3 average 4 not so clear 5 almost/completely unclear

C-5 Can you hear clearly (including wearing hearing aid)?

1 Difficult to hear clearly 2 Need others to raise their voice 3 Can hear clearly

C-6 Do your teeth conditions affect your eating? 1 Yes 0 No

C-7 Do you often feel pain?

1 Yes 0 No [Go to question C8 directly]

C7.1 If you often feel pain, how bad is the pain?

1 not serious 2 moderate 3 serious

C-8 How many times a week do you work out?

1 never 2 less than once 3 one to two times

4 three to five times 5 six times or more

C-9 Do you take any health supplements?

1 Never 2 Occasionally 3 Often

C10 Did you have a medical examination in 2014?

1 Yes 0 No

C-11 Do you have any of the following chronic diseases? (Multiple choice) [Interviewer: Ask item by item]

□ Cataract/glaucoma □ Hypertension □ Diabetes

□ Cardiovascular and cerebrovascular diseases (coronary heart disease/angina pectoris/stroke, etc.) □ Stomach diseases

□ osteoarthropathy (osteoporosis/arthritis/rheumatism/disc disease, etc.)

□ Chronic lung diseases (COPD/tracheitis/emphysema, etc.) □ Asthma

□ Malignant tumors □ Reproductive system diseases

□ Other chronic diseases (please specify) __________ □ None

C12 Were you ill in the past two weeks?

1 Yes 0 No [Go to question C13 directly]

C12.1 What kind of illness do you have this time?

1. It's new in two weeks 2. The acute disease began two weeks ago and continued for two weeks

3. The onset of chronic disease began two weeks ago and continued for two weeks

C12.2 How did you deal with it after you fell ill?

1. Went to see a doctor 2. Did nothing [Go to question C12.4 directly] 3. self-medicated [Go to question C12.5 directly]

C12.3 How many times have you visited the hospital or clinic in the last two weeks?

_ times [Go to question C13 directly]

C12.4 What's the main reason you didn’t do anything with your illness?

(Multiple choice) [Interviewer: Ask item by item]

□ perceived the illness to be light □ financial difficulties □ don't have the time □ limited mobility □Unaccompanied □ Hospital is too far away □ Troublesome for medical treatment □ Other reasons (please specify) __________[Answer this question and skip to C13]

C12.5 Which of the following self-medication measures have you taken?

(Multiple choice) [Interviewer: Ask item by item]

□ Buy your own medicine □ Use traditional methods of treatment □ Use health care and rehabilitation equipment□ Others (please specify) __________

C13 Where do you usually see your doctor?

[Interviewer: Interviewee may have visited multiple medical and health institutions, so select the one with the most frequent visits]

1 private clinic 2 clinics/stations 3 community health service centers 4 township/sub-district health centers 5 county/city/district hospitals 6 city/prefecture level hospitals 7 provincial hospitals 8 others (please specify) __________

C13.1 How far away is the health care facility you visit most from your home?

1. less than 1 km

2. 1to 2 km

3 .3 to 5 km

4 .5 km and above

C-14 Have you encountered any of the following problems when visiting a hospital or clinic? (Multiple choice) [Interviewer: Ask item by item]

□ The waiting time is too long □ The formalities are too tedious □ The barrier-free facilities are not available □ Can’t be hospitalized in time□ The service attitude is not good □ The charge is too high □ Others (please specify) __________

C15 How many times have you been hospitalized in 2014? ______ times

C16 What was your medical expenses in total in 2014? ____RMB

C16.1 Among them, how much you paid as your own expenses (can’t be reimbursed)? _____RMB...

C16.2 How much did your children or other people pay for your medical expenses? ________RMB

C17 What was the expenses paid with your own pocket that you spent at pharmacy in 2014? _____RMB...

C18 Which of the following health insurance/benefits do you have?

(Multiple choice) [Interviewer: Ask item by item]

□ Basic medical insurance for urban employees □ Basic medical insurance for urban residents □ New rural cooperative medical insurance□ Basic medical insurance for urban and rural residents (urban residents' basic medical insurance is integrated with the new rural cooperative medical insurance) □ Serious illness insurance for urban and rural residents □ large medical subsidies for employees□ publicly funded medical care □ Others (please specify) __________ □ None

C18.1 Do you think it is convenient to reimburse medical expenses?

1 very convenient 2 relatively convenient 3 average

4 relatively inconvenient 5 very inconvenient

C19 Do you have commercial health insurance?

1 Yes 0 No

C20 How do you feel about your overall health?

1. very good 2 relatively good 3 average
2. 4 relatively poor 5 very poor

D． Status of health care services

[Interviewer: Next, we'd like to know about your health care services.]

D-1 Which of the following daily activities do you do?

| Daily activities | Can do it | Have some difficulties | Can't do it |
| --- | --- | --- | --- |
| 1 Feeding | 1 | 2 | 3 |
| 2 Dressing | 1 | 2 | 3 |
| 3 Toileting | 1 | 2 | 3 |
| 4 Getting in or out of bed | 1 | 2 | 3 |
| 5. Walking indoors | 1 | 2 | 3 |
| 1. Having a bath | 1 | 2 | 3 |
| 7 Cooking | 1 | 2 | 3 |
| 8 Doing laundry | 1 | 2 | 3 |
| 9 Sweeping the floor | 1 | 2 | 3 |
| 10 Shopping | 1 | 2 | 3 |
| 11 Going up and down the stairs | 1 | 2 | 3 |
| 12 Taking a bus | 1 | 2 | 3 |
| 13 Lifting a weight of 5 kg | 1 | 2 | 3 |
| 14 Using the telephone | 1 | 2 | 3 |
| 15.Manage personal finances | 1 | 2 | 3 |

D-2 Many older people have incontinence, do you have it?

(Multiple choice) [Interviewer: Ask item by item]

□incontinent of bowel □incontinent of bladder □ none

D-3 Are you using any of the following accessories now?

(Multiple choice) [Interviewer: Ask item by item]

□ Reading glasses □ hearing aids □ dentures □ crutches □ wheelchair □ blood pressure monitor

□ blood glucose monitor □ adult diapers/nursing mats □ massage appliances □ smart wearables□ nursing bed □ others (please specify) __________ □ none

D-4 Do you need someone to take care of your daily life currently?

1 Yes 0 No [Go to question D5 directly]

D4.1 Do you have any caregivers?

1Yes 0 No [Go to question D5 directly]

D4.2 Who is your primary caregiver?

1 spouse 2 son 3 daughter-in-law 4 daughter 5 son-in-law

6 grandchildren 7 other relatives 8 friends/neighbors 9 volunteers

10 domestic service personnel (personal care assistant, hourly workers, etc.)

11 personnel from medical institutions

12 nursing home staff 13 community workers 14 others (please specify) _________

D4.3 How old is she/he (the primary caregiver)? Age of the___...

D-5 Are there any other older people in your family who need to be taken care of?

1Yes 0 No [Go to question D6 directly]

D5.1 Who is taking care of him/her now?

1 the interviewee 2 others (please specify) __________

D-6 If it is necessary, where would you like the most to receive care?

1 at home [Go to question D7] 2 at the community during daytime and go back home at night [Go to question D7] 3 in a nursing home 4 depends on situations

D6.1 If you live in an assisted-living facility, what is the maximum monthly expenses you (and your family) can afford?

1. below 1000 yuan 2 .1000-1999 yuan 3. 2000-2999 yuan

4. 3000-3999 yuan 5. 4000-4999 yuan 6. 5000 yuan and above

D7 Your need, knowledge and utilization of the following community service programs for older adults: [Interviewer: item by item inquiry]

| Service | Do you need it | Is it provided | Have you ever used it |
| --- | --- | --- | --- |
| 1.Meal assistance service | 1 Yes 0 No | 1 Yes 2 No  3 Don’t know | 1 Yes 0 No |
| 2. Assisted Bathing services | 1 Yes 0 No | 1 Yes 2 No  3 Don’t know | 1 Yes 0 No |
| 3. Housework assistance | 1 Yes 0 No | 1 Yes 2 No  3 Don’t know | 1 Yes 0 No |
| 4. At your door healthcare | 1 Yes 0 No | 1 Yes 2 No  3 Don’t know | 1 Yes 0 No |
| 5 Care during daytime | 1 Yes 0 No | 1 Yes 2 No  3 Don’t know | 1 Yes 0 No |
| 6 Rehabilitation nursing care | 1 Yes 0 No | 1 Yes 2 No  3 Don’t know | 1 Yes 0 No |
| 7. Rental service of assisted devices for older adults | 1 Yes 0 No | 1 Yes 2 No  3 Don’t know | 1 Yes 0 No |
| 8.Health education services | 1 Yes 0 No | 1 Yes 2 No  3 Don’t know | 1 Yes 0 No |
| 9.Psychological consultation/ Casual chatting | 1 Yes 0 No | 1 Yes 2 No  3 Don’t know | 1 Yes 0 No |

E. Economic status

[Interviewer: Next, we want to ask some basic questions about the financial situation of you and your family.]

E-1 Have you gone through the retirement formalities?

1 Yes 2 No [Go to question E2] 3 Not applicable (Never had a formal job) [Go to question E2]

E1.1 How old were you when you retired? Age of the_____...

E1.2 Did you do early retirement? 1 Yes 2 No

E1.3 What is the type of your workplace before retirement?

1 Party and government 2 public institutions 3 state-owned enterprises 4 collective enterprises 5 private companies 6 foreign-funded enterprises 7 military 8 rural collectives 9 others (please specify) __________

E-2 Are you still employed in a paid job (including working, doing business, etc.)?

1 Yes 0 No [Go to question E2.3]

E2.1 How did you get the job?

1 personal relationship 2 company re-employment 3 job market 4 government aid 5 self-employed 6 others (please specify) __________

E2.2 What was your income last month from the job above? ________RMB

E2.3 Are you willing to be employed in a paid job (including working, doing business, etc.)? 1 Yes 0 No

E-3 * (Rural) Are you engaged in agriculture, forestry, animal husbandry, sideline fishing and other economic activities?

1 Yes 0 No. [Go to question E4]

E3.1What was your net income from the above economic activities in 2014? ________RMB...

E-4 Have you and your spouse saved any money for retirement?

1 Yes 0 No. [Go to question E4]

E4.1 How much is the saved money? _____RMB...

E-5 Do you have any of the following monthly income currently?

[Interviewer: ask item by item, if there is, fill in the specific amount, if there is no, fill in 0]

E5.1 pension ____yuan E5.2 Survivors' benefits ____ yuan E5.3 occupational annuity / enterprise annuity ___________ yuan E5.4 Commercial pension ________ yuan

E5.5 allowance for the oldest old ________ yuan E5.6 Pension service subsidy__________ yuan

E5.7 Nursing care subsidy________ yuan E5.8 Minimum living allowance________ yuan

E5.9 Five insurance subsidy / unemployment benefits _______ yuan

E5.10 Reward (special) subsidy for family planning ________ yuan

E5.11 Other Social Security benefits (please specify)__________，__________ yuan

E-6 Do you and your wife have the following income in 2014? [Interviewer: ask item by item, if there is, fill in the specific amount, if there is no, fill in 0]

E6.1 The income from rental properties is ________ yuan

E6.2 The income from interest is ________ yuan

E6.3 The income from * (rural) land leasing/contracting is ______yuan

E6.4 The income from welfare/collective subsidy/bonus of old workplace is _________ yuan

E6.5 The money gifted by children (grandchildren) (including actual presents) ________ yuan

E6.6 The money gifted by other relatives ________ yuan (including actual presents)

E-7 Which of the following investment and financial activities are you engaged in now?

(Multiple choice) [Interviewer: Ask item by item]

□ Treasury bonds/bonds □ stocks □ funds □ foreign exchange □ precious metals

□ Other financial products □ Other (please specify) __________ □ None [Go to question E8]

E7.1 If there are any financial assets selected above, the total amount is ________ in ten thousand yuan

E-8 Do you (or your spouse) own your home?

1 Yes 0 No. [Go to question E9]

E8.1 What is the number of properties that you own? __...

E8.2 What is the approximate value of these properties currently?

________in ten thousand yuan...

E8.3 Would you be willing to sell/rent/mortgage your house for a pension?

1 yes, 2 no, 3 depends

E9 What kind of housing do you live in now?

1. Owned property 2. Children's property

3. Grandchildren's property 4 Renting public housing

5 Renting private housing 6 staying at other’s house 7 Others (please specify) __________

E10 On average, your monthly expenses spent on daily living:

[Interviewer: ask item by item, if there is, fill in the specific amount, if there is no, fill in 0]

E10.1 The expenditure on personal items (including tobacco and alcohol, cosmetics, toiletries, etc.) is ________ yuan

E10.2 The expenditure on transportation is ________ yuan

E10.3 The expenditure on communication is ________ yuan

E10.4 The expenditure on paying personal care assistant/hourly worker/caregiver is_yuan

E10.5 The expenditure on health care (beauty salons, health care products, massage, etc.) is ________ yuan

E10.6 The expenditure on recreation, sports and entertainment (watching movies, purchasing books and newspapers, etc.) is ________ yuan

E11 Your personal expenses in 2014: [Interviewer: ask item by item, fill in the specific amount if there is any, or fill in 0 if there is no]

E11.1 The expenditure on purchasing clothes, shoes and hats is ________ yuan

E11.2 The expenditure on tourism is ________ yuan

E11.3 Gifted ___yuan to children/grandchildren

E11.4 The expenditure on buying assistive equipment (dentures, wheelchair, hearing aid, etc.) is ________ yuan

E12 Expenditure information of you and your spouse in 2014:

[Interviewer: ask item by item, if there is any, fill in the specific amount; if there is no, fill in 0]

E12.1 Expenditure on housing rental is_______________ yuan

E12.2 Expenditure on heating is ____________ yuan

E12.3 Property management fee is ______________ yuan

E12.4 Expenditure on the house purchasing/interior finishing is________ million yuan

E12.5 Expenditure on purchasing furniture and appliances is ________ yuan

E12.6 Expenditure on buying vehicles is __________ yuan

E12.7 Expenditure on buying expensive jewelries is ____________ yuan

E13 The average monthly expenditure on food (board) in your family is _____________ yuan

E14 The total expenditure of your family in 2014 is ____million yuan

E15 In 2014, the total income of your family is _______million yuan

E16 How much do you or your family own debts? ____ yuan

E-17 Do you think your grandchildren/children are "mooching off" their parents?

1 Yes 0 No.

E18 What type of situations would you consider your financial situation to be?

1 very wealthy 2 relatively wealthy 3 basically enough 4 relatively difficult 5 very difficult

F． Conditions of livable environment

[Interviewer: Next, we want to know about your livable environment]

F-1 When was the house you are living in built?

1. Before liberation 2.1950s-1960s 3. 1970s-1980s

4. In the 90 s 5. after 2000s

F-2 What is the total floor area of the place you are living at?

__________ square meters

F-3 Do you have a private room (with your spouse)? 1 Yes 0 No

F-4 Do you have the following living facilities in your present housing? (Multiple choice) [Interviewer: Asking by item]

□ tap water □ gas/natural gas/biogas □ heating

□ Indoor toilets □ Bath/shower facilities □ None

F-5 Do you have any of the following electronics and household appliances in your current housing? (Multiple choice) [Interviewer: Ask item by item]

□ landline phone □ mobile phone for elderly □ smartphone □ basic mobile phone □ computer □ TV□ washing machine □ air conditioner □ refrigerator □ air purifier □ water purification device □ None

F-6 Have you had any falls this year?

1 Yes 0 No [Go to question F7]

F6.1 Where did you fall last time?

1 bedroom 2 bathroom 3 living room 4 kitchen

5 balcony 6 doorsill 7 stairs

8 courtyards 9 roads 10 on transportation 11 shopping places 12 fitness places

13 parks 14 workplace 15 other locations (please specify) _____

F6.2 What were the consequences of your fall?

1 no injury 2 minor injuries, no need of medical attention 3 serious injuries, requiring medical attention 4 serious injuries, long term in bed

F-7 Which of the following conditions do you have in your current residence? (Multiple choice) [Interviewer: Ask item by item]

□ dim light □ stumbling doorsills or uneven floor □ no handrails □ slippery floor

□ Door doesn't work properly □ Toilet/bathroom doesn't work well □ No calling/alarm facilities

□ Noisy □ Others (please specify) __________ □ Everything is fine, no problem exists

F-8 Are you satisfied with your present living conditions?

1 Satisfied 2 Average 3 Not satisfied

F-9 How many years have you lived in this community (village/residence) _____ years

F-10 What kind of relationship do you have with your neighbors?

1 Don't know 2 Just say hello

3 Communicate frequently 4 Help each other when necessary

F-11 Are you satisfied with any of the following aspects in your community (village/residence)? (Multiple choice)

□ Signage □ Road/street lighting □ Traffic conditions □ Household facilities

□ Fitness place □ Public restroom □ Green areas □ Public security

□ The atmosphere of respecting the elderly □ none is satisfied

G． Social participation

[Interviewer: Next, we want to know about your social participation]

G-1 Do you often participate in any of the following volunteer activities? (Multiple choice)

□ Neighborhood watch □ Helping mediate neighborhood disputes □ Keeping community hygiene □ Helping neighbors □ Caring about educating the next generation (not including educating your grandchildren)

□ Participating in cultural and scientific promotion activities □ None

G-2 Which of the following organizations or groups have you joined?

(Multiple choice) [Interviewer: Ask item by item]

□ Community security group □ Mediation committee

□ Non-profit organizations (volunteer/charity, etc.) □ Recreational and sports organizations (painting/singing/dancing, etc.) □ Folk culture organizations □ Professional and technical groups or organizations □Cooperative organizations for the elderly (voluntary pension groups/economic organizations for the elderly)

□ Other organizations (please specify) __________ □ None

G-3 Have you attended any of the following clan/clan activities?

(Multiple choice) [Interviewer: Ask item by item]

□ Repair family tree/genealogy □ Take part in ancestor worship activities □ Take part in charity activities organized by the family/clan□ Help to mediate intra-clan or inter-clan disputes □ Other activities (please specify) __________ □None

G-4 Are you a member of a senior association?

1 Yes 0 No. [Go to question G4.3].

G4.1 Are you satisfied with the activities organized by the senior association?

1 Very Satisfied 2 Relatively satisfied 3 Average

4 Relatively unsatisfied 5 Very Unsatisfied

G4.2 What kind of activities would you like the senior association to organize?

(Multiple choice) [Interviewer: Ask item by item]

□ learning/recreational activities □ activities aiming to help those older adults with difficulties □ family activities involving both the younger and older adults □ protection of the rights and interests of the elderly □ voluntary activities □ profit-making activities□ participate in community public affairs □ others (please specify) __________ □ no suggestion [Go to question G5]

G4.3 What are the main reasons that you don't join a senior association?

(Multiple choice) [Interviewer: Ask item by item]

□ Not association established □ Not interested □ No time □ Health problems

□ Family members do not support □ Others (please specify) ___

G-5 Are you willing to help other older adults in your community who are in need? 1 Yes 0 No

G-6 Did you take part in the last community election? 1 Yes 0 No

G-7 Are you concerned about community affairs being publicized?

1 Yes 2 No 3 It doesn't matter

G-8 Have you ever been asked on important projects in your community? 1 Yes 0 No

G-9 Have you made suggestions to the community? 1 Yes 0 No

G-10 Do you care about state affairs? 1 Yes 0 No

H． Rights Protection Status

[Interviewer: Next, we want to know about your rights protection status]

H-1 Do you know the <Law on the Protection of the Rights and Interests of the Elderly>?

1 Yes 0 No

H-2 Have you gotten a privilege card for senior citizens? 1 Yes 0 No

H-3 Have you ever enjoyed any of the following preferential treatments for the elderly? (Multiple choice) [Interviewer: Ask item by item]

□ Free physical examination □ Discount or no fees for general outpatient service □Discount or free for public transportation □ Discount or free for park entrance □ Discount or free for tourist attractions □ Discount or free for places such as museums and public libraries □None

H-4 Has your family treated you in any of the following ways this year?

(Multiple choice) [Interviewer: Ask item by item]

□ Did not provide your basic living expenses when you asked for □The accommodation provided to you is in poor condition □You are not fed well/poorly fed □No support of medical treatment for you □ Did not take care of you when you needed it □ encroaching on your property □ Didn’t come to visit /talk to you for a long time□ often beat and scold you * (only for widowed or divorced interviewee) □ interfere your remarriage□ Other actions (please specify) ___□ None [Go to question H5]

H4.1 If there were any of the above situations happened, what measures have you taken to resolve it? (Multiple choice)

□ Knuckled under □ Asked relatives/clans to help mediate □Went to the neighborhood committee for help. □Went to the seniors association for help□ Asked the family’s workplace for mediation □ lawsuit/asked the judicial authority to solve

□ Reported to the media □ Others (please specify) __________

H-5 Which of the following situations have you encountered so far this year? (Multiple choice) [Interviewer: Ask item by item]

□ be cheated □ be robbed □ be stolen

□ be beaten/threatened □ others (please specify) _______□ none

H-6 Have you received any legal aid this year? 1 Yes 0 No

H-7 Do you think your legal rights and interests are properly protected? 1 Yes 0 No

I. Spiritual and cultural life

[Interviewer: Next, we want to know about your spiritual and cultural life]

I-1 Do you often do any of the following activities?

(Multiple choice) [Interviewer: Ask item by item]

□Watch TV/listen to the radio □ read books/newspapers□ go to the cinema/theatre □go for a walk/jog, etc □ Tai Chi/Health exercises etc. □Dancing (Public square dancing/Yangko dancing) □Play baseball/table tennis/badminton etc. □Play mahjong/cards/chess, etc

□ gardening □ have pets

□ Fishing/Calligraphy/Photography/Collection □ Others (please specify) __________□None

I-2 Do you often surf the Internet?

1 Yes 0 No [Go to question I3]

I2.1 Do you do any of the following activities when you surf the Internet?

(Multiple choice) [Interviewer: Ask item by item]

□ watching news □ watching movies and TV shows □ chatting □ shopping□ Playing games □ buying stocks □ Others (please specify) __________

I-3 Have you attended a college/school for older adults (including online education for older adults)? 1 Yes 0 No

I-4 Do you have any of the following public places available near your home? How often do you go to those places?

[Interviewer: Ask item by item. If the interviewee answered "no" or "don't know", no need to ask further about the frequency of going to the place]

| Public Place/Facility | Available or not | How often do you go |
| --- | --- | --- |
| 1.Square | 1 Yes 2 No  3 Don’t know | 1 Never 2 Occasionally  3 Usually |
| 2.Park | 1 Yes 2 No  3 Don’t know | 1 Never 2 Occasionally  3 Usually |
| 3.Fitness centers | 1 Yes 2 No  3 Don’t know | 1 Never 2 Occasionally  3 Usually |
| 4.Senior citizens’ activity center | 1 Yes 2 No  3 Don’t know | 1 Never 2 Occasionally  3 Usually |
| 5.Library | 1 Yes 2 No  3 Don’t know | 1 Never 2 Occasionally  3 Usually |

I-5 How long do you usually spent your daily time in doing each of the activities lised below？

| Activity type | Time |
| --- | --- |
| 1.Paid work /business activity | ___________________hours |
| 2.Housework | ___________________hours |
| 3.Watching television | ___________________hours |
| 4.Reading books and newspapers | ___________________hours |
| 5.Other leisure activities | ___________________hours |
| 6.Napping | ___________________hours |

I-6 Do you have any plans to travel in the coming year?

1 Yes 2 No 3 Not sure

I-7 What is your religion?

1 None 2 Buddhism 3 Islam

4 Christianity 5 Catholicism 6 Taoism

7 Other religions (please specify) __________

I-8 Have any of the following situations ever happened to you in daily life?

(Multiple choice) [Interviewer: Ask item by item]

□Perceived your friends or relatives as strangers when looking at their faces □ often cannot remember the names of relatives or friends □Unable to find my way home after going out □ I often forget to take my keys□I often forget to turn off the stove□None

I-9 Do you feel lonely? 1 Often 2 Sometimes 3 Never

I-10 During the past week, did you have any of the following feelings?

(Multiple choice) [Interviewer: Ask item by item]

□Felt happy most of the time. □Felt irritable and restless all day

□Felt upset all the time. □Thinking it's good to be alive

I-11 How old do you feel? Age of the___

I-12 A few older adults committed suicide for various reasons. What do you think of that?

1. Cherish life 2. let it go naturally 3. one has the right to give up his /her own life

I-13 Do you agree with the following statements?

(Multiple choice) [Interviewer: Ask item by item]

□Older adults should devote their remaining energy and participate in social development □Older adults should enjoy life and be supported by their families and society □Older adults are a burden on their families □Older adults are a burden on the society □Older adults are valuable assets to the country and society □Older adults should be independent and do their best to not cause troubles to their children and society

I-14 In general, do you think you are happy?

1 very happy 2 relatively happy 3 average

4 relatively unhappy 5 very unhappy

***************************************************************************

[Interviewer: For the convenience of the institute to verify the information of this interview, please leave your name and contact information]

Signature of interviewee ___________； phone number __________

Signature of the third-party respondent ______； phone number __________

This is the end of the interview, thank you for your support and cooperation!

J． Interview Notes

[Interviewer: After the interview, please fill the following questions according to the interview situation]

J1 What kind of residence is the interviewee living at now?

1 apartment 2 bungalow 3 house built with mud bricks 4 others (please specify) ____

J2 If it is an apartment, what floor is the interviewee living on? ______F

J3 If it's an apartment, is there an elevator?

1 Yes 0 No

J4 Were there any other people present during the interview?

1 Yes 0 No

J5 Were there any respondents answer questions on behalf of the interviewee?

1 Yes 0 No [Go to question J8]

J6 Respondent’s the relationship to the interviewee

1 spouse 2 children 3 grandchildren 4 others (please specify) ______

J7 The reason for having the respondent: (multiple choice)

□Unable to answer because of deafness. □ Unable to answer because of dementia□ Unclear responses □ Hearing impairment□ Unavailable for interview due to illness □ Others (please specify) __________

J8 Judge the health condition of the interviewee:

1 very healthy 2 relatively healthy 3 average 4 relatively unhealthy 5 very unhealthy

J9 Judge the self-caring ability of the interviewee:

1 completely independent 2 partially independent 3 completely dependent

**Supplement 3. Supplemental figures and tables of results**

**Supplement 3-figure S1. Flowchart of data quality control for the 4th Survey of the Aged Population in Urban and Rural China (SSAPUR) study participants.**

**
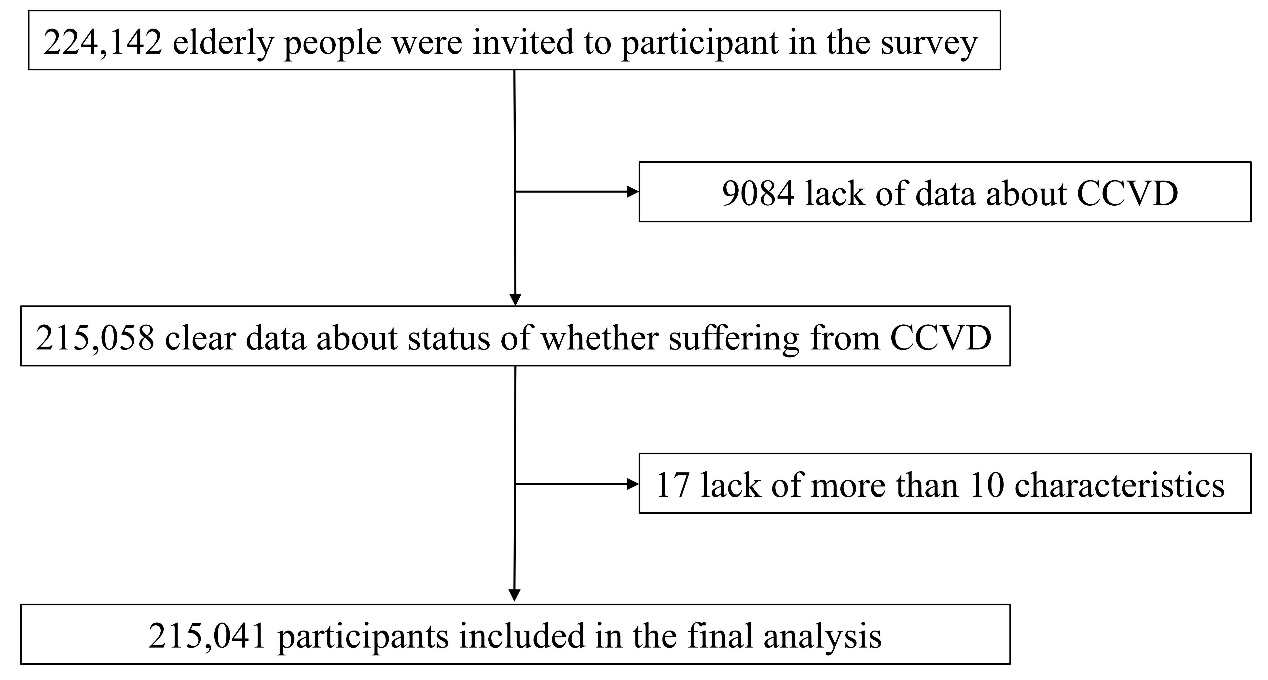
**

**Supplement 3-figure S2. Geographic distribution of the prevalence of CCVD in 31 selected provinces/municipalities in the 4th Survey of the Aged Population in Urban and Rural China (SSAPUR) study. The numbers in parentheses indicate the prevalence of CCVD in each location.**


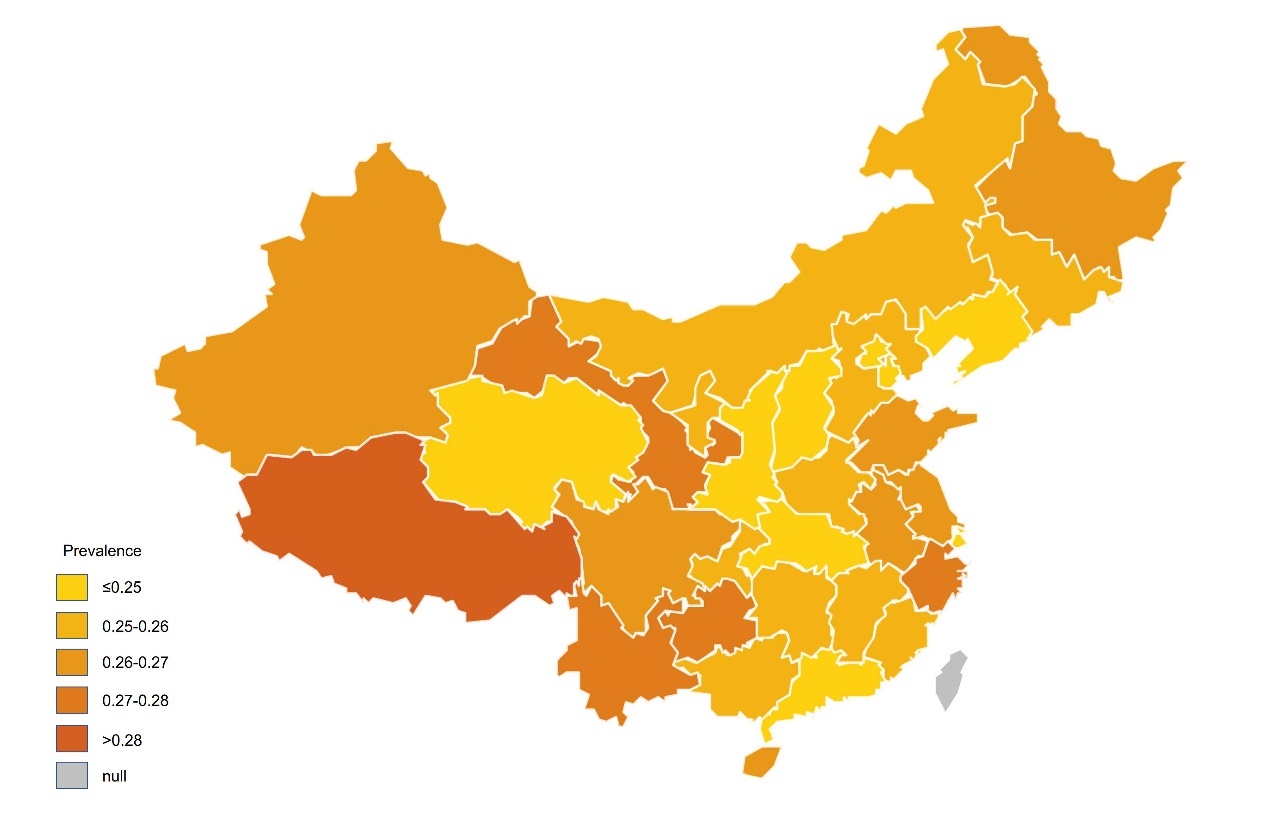


**Supplement 3-figure S3. Geographic distribution of the prevalence of CCVD in 31 selected provinces/municipalities in the 4th Survey of the Aged Population in Urban and Rural China (SSAPUR) study Numbers in parentheses indicate the sample size from each location.**


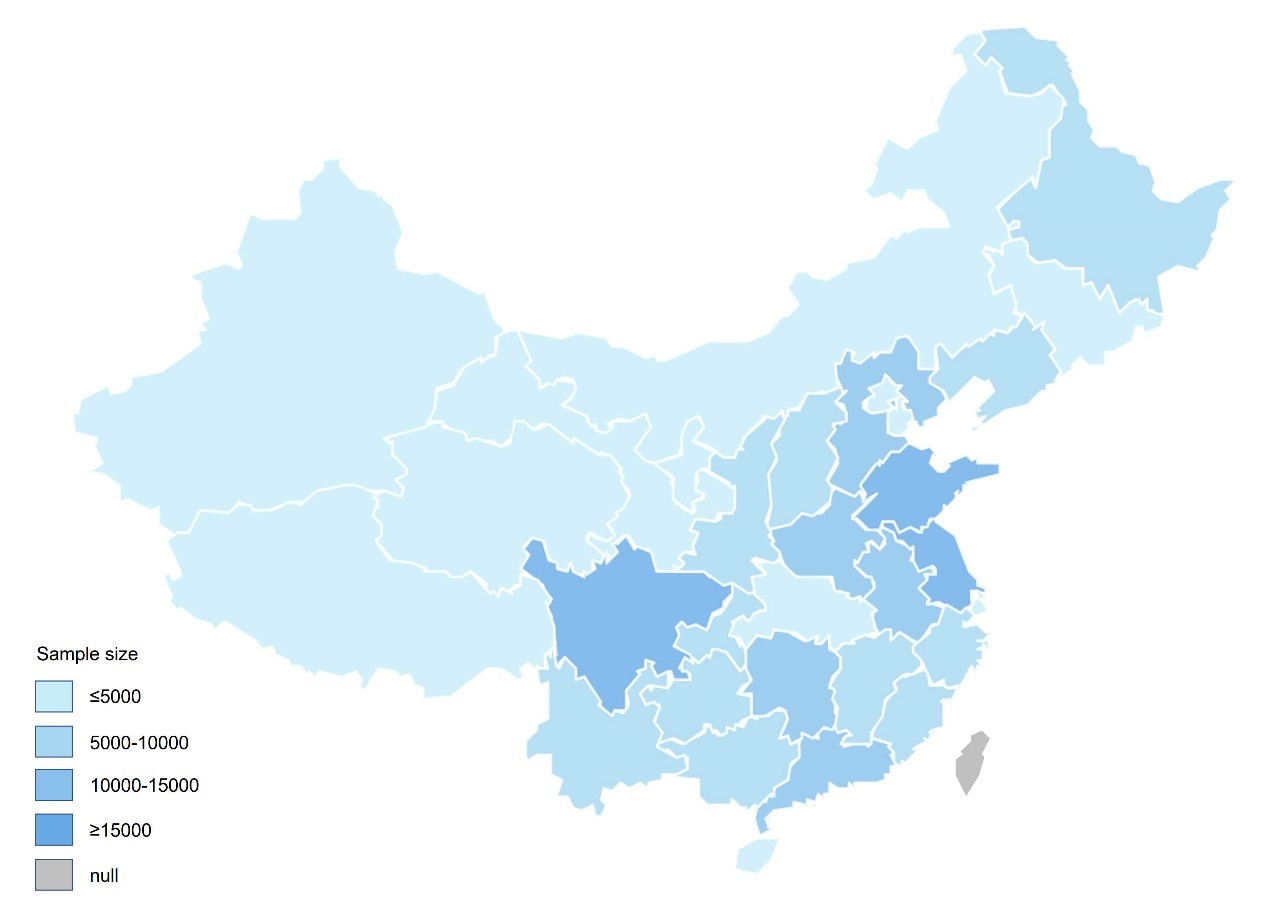


**Supplement 3-table S1.**  **Univariate and** **multivariate logistic analysis for** **cardiac-cerebral vascular disease.**

| Factor | Univariate | | multivariate | |
| --- | --- | --- | --- | --- |
|  | OR(95%CI） | P-value | OR(95%CI） | P-value |
| Urban and rural |  | <0.001 | 1.062(1.040-1.085) | <0.001 |
| Town | 1 |  |  |  |
| Rural area | 1.127(1.105-1.149) | <0.001 |  |  |
| Age（years） |  | <0.001 | 0.986(0.979-0.992) | <0.001 |
| 60-64 | 1 |  |  |  |
| 65-69 | 0.943(0.918-0.968) | <0.001 |  |  |
| 70-74 | 0.984(0.956-1.013) | 0.288 |  |  |
| 75-79 | 0.979(0.949-1.011) | 0.192 |  |  |
| 80-84 | 0.996(0.96-1.033) | 0.834 |  |  |
| ≥85 | 1.034(0.989-1.082) | 0.144 |  |  |
| Sex |  | <0.001 | 0.894(0.876-0.913) | <0.001 |
| Female | 1 |  |  |  |
| Male | 0.842(0.826-0.859) | <0.001 |  |  |
| Education level |  | <0.001 | 0.911(0.901-0.922) | <0.001 |
| Have not attended school (including literacy classes) | 1 |  |  |  |
| Primary school (including private school) | 0.779(0.761-0.797) | <0.001 |  |  |
| Junior high school | 0.614(0.596-0.632) | <0.001 |  |  |
| High school/secondary school/vocational high school | 0.787(0.756-0.82) | <0.001 |  |  |
| College | 0.731(0.68-0.785) | <0.001 |  |  |
| Bachelor degree and above | 0.768(0.699-0.845) | <0.001 |  |  |
| Marriage |  | <0.001 | 1.022(1.001-1.044) | 0.044 |
| Married | 1 |  |  |  |
| Widowed | 1.107(1.083-1.132) | <0.001 |  |  |
| Divorce | 1.017(0.914-1.131) | 0.757 |  |  |
| Never married | 1.095(1.012-1.186) | 0.025 |  |  |
| Live alone |  | <0.001 | 1.032(0.998-1.067) | 0.068 |
| No | 1 |  |  |  |
| Yes | 1.090(1.060-1.121) | <0.001 |  |  |
| Smoking |  | 0.022 | 1.063(1.022-1.106) | 0.003 |
| No | 1 |  |  |  |
| Yes | 1.046(1.006-1.088) | 0.022 |  |  |
| Drinking |  | <0.001 | 0.919(0.878-0.963) | <0.001 |
| Never or occasionally | 1 |  |  |  |
| 1-2 times a week | 0.796(0.676-0.936) | 0.006 |  |  |
| At least 3 times a week | 0.755(0.677-0.841) | <0.001 |  |  |
| Often drunk | 0.818(0.619-1.08) | 0.156 |  |  |
| Sleep |  | <0.001 | 1.044(1.014-1.076) | 0.004 |
| That's good | 1 |  |  |  |
| Better | 1.112(1.003-1.232) | 0.043 |  |  |
| General | 1.312(1.204-1.429) | <0.001 |  |  |
| Relatively poor | 1.19(1.064-1.331) | 0.002 |  |  |
| Very bad | 1.257(1.047-1.508) | 0.014 |  |  |
| Workout |  | <0.001 | 0.996(0.990-1.003) | 0.247 |
| Never exercise | 1 |  |  |  |
| Less than once | 0.978(0.932-1.025) | 0.354 |  |  |
| Once or twice | 0.941(0.913-0.97) | <0.001 |  |  |
| Three to five times | 0.933(0.905-0.962) | <0.001 |  |  |
| Six times and above | 0.881(0.859-0.904) | <0.001 |  |  |
| Hypertension |  | 0.121 |  |  |
| No | 1 |  |  |  |
| Yes | 1.016(0.996-1.036) | 0.121 |  |  |
| Diabetes |  | 0.945 |  |  |
| No | 1 |  |  |  |
| Yes | 0.999(0.965-1.034) | 0.945 |  |  |
| Stomach disease |  | 0.172 |  |  |
| No | 1 |  |  |  |
| Yes | 1.018(0.992-1.044) | 0.172 |  |  |
| Medical insurance |  | 0.933 |  |  |
| No | 1 |  |  |  |
| Yes | 0.996(0.9-1.102) | 0.933 |  |  |
| Elderly people in your home who require care |  | 0.652 |  |  |
| No | 1 |  |  |  |
| Yes | 0.993(0.964-1.023) | 0.652 |  |  |
| Gainful employment |  | 0.549 |  |  |
| No | 1 |  |  |  |
| Yes | 0.99(0.959-1.023) | 0.549 |  |  |
| Economic status |  | <0.001 | 0.992(0.979-1.006) | 0.273 |
| Very generous | 1 |  |  |  |
| Relatively ample | 1.032(0.943-1.13) | 0.489 |  |  |
| Basically enough | 1.054(0.966-1.151) | 0.239 |  |  |
| Tougher | 1.129(1.032-1.234) | 0.008 |  |  |
| Very difficult | 1.104(1.000-1.219) | 0.049 |  |  |
| Not participating in public welfare activities |  | 0.007 | 1.006(0.986-1.026) | 0.569 |
| No | 1 |  |  |  |
| Yes | 1.027(1.007-1.047) | 0.007 |  |  |
| No external abuse |  | 0.57 |  |  |
| No | 1 |  |  |  |
| Yes | 0.987(0.942-1.034) | 0.57 |  |  |
| Non-spiritual cultural life |  | 0.000 | 1.010(0.973-1.048) | 0.601 |
| No | 1 |  |  |  |
| Yes | 1.102(1.064-1.141) | 0.000 |  |  |

**Supplement 3-table S2. The analysis of the relationship between parameters and cardiac-cerebral vascular disease by gender stratification**

| Factor | All | | | Female | | | Male | | |
| --- | --- | --- | --- | --- | --- | --- | --- | --- | --- |
|  | Without CCVD | With CCVD | **P-value** | Without CCVD | With CCVD | **P-value** | Without CCVD | With CCVD | **P-value** |
| Urban and rural |  |  | <0.001 |  |  | <0.001 |  |  | <0.001 |
| Town | 84069 | 27871 |  | 44049 | 15621 |  | 40020 | 12250 |  |
| Rural area | 75059 | 28042 |  | 37321 | 15358 |  | 37738 | 12684 |  |
| Age（years） |  |  | <0.001 |  |  | 0.267 |  |  | <0.001 |
| 60-64 | 52232 | 18681 |  | 26257 | 9866 |  | 25975 | 8815 |  |
| 65-69 | 37934 | 12789 |  | 18726 | 7019 |  | 19208 | 5770 |  |
| 70-74 | 26448 | 9312 |  | 13279 | 5205 |  | 13169 | 4107 |  |
| 75-79 | 20834 | 7297 |  | 10802 | 4169 |  | 10032 | 3128 |  |
| 80-84 | 13586 | 4840 |  | 7462 | 2856 |  | 6124 | 1984 |  |
| ≥85 | 8094 | 2994 |  | 4844 | 1864 |  | 3250 | 1130 |  |
| Education level |  |  | <0.001 |  |  | <0.001 |  |  | <0.001 |
| Have not attended school (including literacy classes) | 43934 | 19168 |  | 33310 | 14940 |  | 10624 | 4228 |  |
| Primary school (including private school) | 66476 | 22583 |  | 30721 | 11244 |  | 35755 | 11339 |  |
| Junior high school | 31952 | 8556 |  | 11338 | 3038 |  | 20614 | 5518 |  |
| High school/secondary school/vocational high school | 11229 | 3858 |  | 4346 | 1193 |  | 6883 | 2665 |  |
| College | 3237 | 1032 |  | 941 | 296 |  | 2296 | 736 |  |
| Bachelor degree and above | 1739 | 583 |  | 446 | 180 |  | 1293 | 403 |  |
| Marriage |  |  | <0.001 |  |  | <0.001 |  |  | <0.001 |
| Married | 116240 | 39733 |  | 52531 | 19615 |  | 63709 | 20118 |  |
| Widowed | 39278 | 14864 |  | 28182 | 11141 |  | 11096 | 3723 |  |
| Divorce | 1332 | 463 |  | 532 | 173 |  | 800 | 290 |  |
| Never married | 2278 | 853 |  | 125 | 50 |  | 2153 | 803 |  |
| Live alone |  |  | <0.001 |  |  | <0.001 |  |  | 0.004 |
| No | 138148 | 47970 |  | 69033 | 25975 |  | 69115 | 21995 |  |
| Yes | 20980 | 7943 |  | 12337 | 5004 |  | 8643 | 2939 |  |
| Smoking |  |  | 0.023 |  |  | <0.001 |  |  | 0.235 |
| No | 10871 | 3662 |  | 7926 | 2759 |  | 2945 | 903 |  |
| Yes | 148257 | 52251 |  | 73444 | 28220 |  | 74813 | 24031 |  |
| Drinking |  |  | <0.001 |  |  | 0.763 |  |  | <0.001 |
| Never or occasionally | 156687 | 55249 |  | 81125 | 30893 |  | 75562 | 24356 |  |
| 1-2 times a week | 663 | 186 |  | 89 | 35 |  | 574 | 151 |  |
| At least 3 times a week | 1556 | 414 |  | 140 | 47 |  | 1416 | 367 |  |
| Often drunk | 222 | 64 |  | 16 | 4 |  | 206 | 60 |  |
| Sleep |  |  | <0.001 |  |  | <0.001 |  |  | <0.001 |
| That's good | 2475 | 670 |  | 955 | 293 |  | 1520 | 377 |  |
| Better | 5020 | 1511 |  | 2226 | 762 |  | 2794 | 749 |  |
| General | 148028 | 52560 |  | 75885 | 29136 |  | 72143 | 23424 |  |
| Relatively poor | 3020 | 973 |  | 1906 | 636 |  | 1114 | 337 |  |
| Very bad | 585 | 199 |  | 398 | 152 |  | 187 | 47 |  |
| Workout |  |  | <0.001 |  |  | <0.001 |  |  | 0.012 |
| Never exercise | 76957 | 28256 |  | 41117 | 16531 |  | 35840 | 11725 |  |
| Less than once | 6956 | 2497 |  | 3716 | 1479 |  | 3240 | 1018 |  |
| Once or twice | 20499 | 7083 |  | 10527 | 3989 |  | 9972 | 3094 |  |
| Three to five times | 19618 | 6721 |  | 9891 | 3516 |  | 9727 | 3205 |  |
| Six times and above | 35098 | 11356 |  | 16119 | 5464 |  | 18979 | 5892 |  |
| Hypertension |  |  | 0.121 |  |  | 0.413 |  |  | 0.920 |
| No | 100619 | 35149 |  | 49266 | 18673 |  | 51353 | 16476 |  |
| Yes | 58509 | 20764 |  | 32104 | 12306 |  | 26405 | 8458 |  |
| Diabetes |  |  | 0.951 |  |  | 0.946 |  |  | 0.277 |
| No | 145270 | 51049 |  | 73426 | 27959 |  | 71844 | 23090 |  |
| Yes | 13858 | 4864 |  | 7944 | 3020 |  | 5914 | 1844 |  |
| Stomach disease |  |  | 0.173 |  |  | 0.973 |  |  | 0.427 |
| No | 130855 | 45835 |  | 65447 | 24914 |  | 65408 | 20921 |  |
| Yes | 28273 | 10078 |  | 15923 | 6065 |  | 12350 | 4013 |  |
| Medical insurance |  |  | 0.957 |  |  | 0.921 |  |  | 0.689 |
| No | 157676 | 55405 |  | 80583 | 30677 |  | 77093 | 24728 |  |
| Yes | 1452 | 508 |  | 787 | 302 |  | 665 | 206 |  |
| Elderly people in your home who require care |  |  | 0.658 |  |  | 0.356 |  |  | 0.427 |
| No | 140396 | 49371 |  | 72238 | 27563 |  | 68158 | 21808 |  |
| Yes | 18732 | 6542 |  | 9132 | 3416 |  | 9600 | 3126 |  |
| Gainful employment |  |  | 0.555 |  |  | 0.260 |  |  | 0.084 |
| No | 143172 | 50356 |  | 76798 | 29184 |  | 66374 | 21172 |  |
| Yes | 15956 | 5557 |  | 4572 | 1795 |  | 11384 | 3762 |  |
| Economic status |  |  | <0.001 |  |  | <0.001 |  |  | 0.010 |
| Very generous | 2060 | 678 |  | 922 | 348 |  | 1138 | 330 |  |
| Relatively ample | 23676 | 8045 |  | 11327 | 4162 |  | 12349 | 3883 |  |
| Basically enough | 94030 | 32620 |  | 48384 | 18115 |  | 45646 | 14505 |  |
| Tougher | 32910 | 12225 |  | 17380 | 7005 |  | 15530 | 5220 |  |
| Very difficult | 6452 | 2345 |  | 3357 | 1349 |  | 3095 | 996 |  |
| Not participating in public welfare activities |  |  | 0.007 |  |  | 0.458 |  |  | 0.116 |
| No | 72625 | 25149 |  | 34638 | 13111 |  | 37987 | 12038 |  |
| Yes | 86503 | 30764 |  | 46732 | 17868 |  | 39771 | 12896 |  |
| No external abuse |  |  | 0.569 |  |  | 0.513 |  |  | 0.756 |
| No | 7097 | 2526 |  | 3508 | 1363 |  | 3589 | 1163 |  |
| Yes | 152031 | 53387 |  | 77862 | 29616 |  | 74169 | 23771 |  |
| Non-spiritual cultural life |  |  | <0.001 |  |  | <0.001 |  |  | 0.022 |
| No | 146924 | 51225 |  | 73665 | 27832 |  | 73259 | 23393 |  |
| Yes | 12204 | 4688 |  | 7705 | 3147 |  | 4499 | 1541 |  |

**Supplement 3-table S3. The analysis of the relationship between parameters and cardiac-cerebral vascular disease by** **“urban and rural” stratification.**

| Factor | All | | | Urban | | | Rural area | | |
| --- | --- | --- | --- | --- | --- | --- | --- | --- | --- |
|  | Without CCVD | With CCVD | **P-value** | Without CCVD | With CCVD | **P-value** | Without CCVD | With CCVD | **P-value** |
| Age（years） |  |  | <0.001 |  |  | <0.001 |  |  | <0.001 |
| 60-64 | 52232 | 18681 |  | 26921 | 9248 |  | 25311 | 9433 |  |
| 65-69 | 37934 | 12789 |  | 19794 | 6083 |  | 18140 | 6706 |  |
| 70-74 | 26448 | 9312 |  | 13762 | 4778 |  | 12686 | 4534 |  |
| 75-79 | 20834 | 7297 |  | 11299 | 3619 |  | 9535 | 3678 |  |
| 80-84 | 13586 | 4840 |  | 7731 | 2635 |  | 5855 | 2205 |  |
| ≥85 | 8094 | 2994 |  | 4562 | 1508 |  | 3532 | 1486 |  |
| Sex |  |  | <0.001 |  |  | <0.001 |  |  | <0.001 |
| Female | 81370 | 30979 |  | 44049 | 15621 |  | 37321 | 15358 |  |
| Male | 77758 | 24934 |  | 40020 | 12250 |  | 37738 | 12684 |  |
| Education level |  |  | <0.001 |  |  | <0.001 |  |  | <0.001 |
| Have not attended school (including literacy classes) | 43934 | 19168 |  | 17405 | 7180 |  | 26529 | 11988 |  |
| Primary school (including private school) | 66476 | 22583 |  | 31233 | 9871 |  | 35243 | 12712 |  |
| Junior high school | 31952 | 8556 |  | 20847 | 5916 |  | 11105 | 2640 |  |
| High school/secondary school/vocational high school | 11229 | 3858 |  | 9426 | 3258 |  | 1803 | 600 |  |
| College | 3237 | 1032 |  | 3175 | 1010 |  | 62 | 22 |  |
| Bachelor degree and above | 1739 | 583 |  | 1732 | 582 |  | 7 | 1 |  |
| Marriage |  |  | <0.001 |  |  | <0.001 |  |  | <0.001 |
| Married | 116240 | 39733 |  | 62360 | 20160 |  | 53880 | 19573 |  |
| Widowed | 39278 | 14864 |  | 19991 | 7184 |  | 19287 | 7680 |  |
| Divorce | 1332 | 463 |  | 978 | 321 |  | 354 | 142 |  |
| Never married | 2278 | 853 |  | 740 | 206 |  | 1538 | 647 |  |
| Live alone |  |  | <0.001 |  |  | 0.005 |  |  | <0.001 |
| No | 138148 | 47970 |  | 73836 | 24302 |  | 64312 | 23668 |  |
| Yes | 20980 | 7943 |  | 10233 | 3569 |  | 10747 | 4374 |  |
| Smoking |  |  | 0.023 |  |  | 0.525 |  |  | 0.015 |
| No | 10871 | 3662 |  | 5927 | 1933 |  | 4944 | 1729 |  |
| Yes | 148257 | 52251 |  | 78142 | 25938 |  | 70115 | 26313 |  |
| Drinking |  |  | <0.001 |  |  | <0.001 |  |  | 0.006 |
| Never or occasionally | 156687 | 55249 |  | 82893 | 27595 |  | 73794 | 27654 |  |
| 1-2 times a week | 663 | 186 |  | 329 | 77 |  | 334 | 109 |  |
| At least 3 times a week | 1556 | 414 |  | 748 | 176 |  | 808 | 238 |  |
| Often drunk | 222 | 64 |  | 99 | 23 |  | 123 | 41 |  |
| Sleep |  |  | <0.001 |  |  | <0.001 |  |  | <0.001 |
| That's good | 2475 | 670 |  | 1468 | 360 |  | 1007 | 310 |  |
| Better | 5020 | 1511 |  | 2769 | 826 |  | 2251 | 685 |  |
| General | 148028 | 52560 |  | 78153 | 26171 |  | 69875 | 26389 |  |
| Relatively poor | 3020 | 973 |  | 1396 | 412 |  | 1624 | 561 |  |
| Very bad | 585 | 199 |  | 283 | 102 |  | 302 | 97 |  |
| Workout |  |  | <0.001 |  |  | 0.002 |  |  | <0.001 |
| Never exercise | 76957 | 28256 |  | 29274 | 9893 |  | 47683 | 18363 |  |
| Less than once | 6956 | 2497 |  | 3909 | 1328 |  | 3047 | 1169 |  |
| Once or twice | 20499 | 7083 |  | 11745 | 3987 |  | 8754 | 3096 |  |
| Three to five times | 19618 | 6721 |  | 12662 | 4252 |  | 6956 | 2469 |  |
| Six times and above | 35098 | 11356 |  | 26479 | 8411 |  | 8619 | 2945 |  |
| Hypertension |  |  | 0.121 |  |  | 0.606 |  |  | 0.004 |
| No | 100619 | 35149 |  | 50580 | 16720 |  | 50039 | 18429 |  |
| Yes | 58509 | 20764 |  | 33489 | 11151 |  | 25020 | 9613 |  |
| Diabetes |  |  | 0.951 |  |  | 0.640 |  |  | 0.198 |
| No | 145270 | 51049 |  | 74530 | 24680 |  | 70740 | 26369 |  |
| Yes | 13858 | 4864 |  | 9539 | 3191 |  | 4319 | 1673 |  |
| Stomach disease |  |  | 0.173 |  |  | 0.108 |  |  | 0.447 |
| No | 130855 | 45835 |  | 71339 | 23539 |  | 59516 | 22296 |  |
| Yes | 28273 | 10078 |  | 12730 | 4332 |  | 15543 | 5746 |  |
| Medical insurance |  |  | 0.957 |  |  | 0.099 |  |  | 0.135 |
| No | 157676 | 55405 |  | 83296 | 27645 |  | 74380 | 27760 |  |
| Yes | 1452 | 508 |  | 773 | 226 |  | 679 | 282 |  |
| Elderly people in your home who require care |  |  | 0.658 |  |  | 0.253 |  |  | 0.440 |
| No | 140396 | 49371 |  | 73795 | 24537 |  | 66601 | 24834 |  |
| Yes | 18732 | 6542 |  | 10274 | 3334 |  | 8458 | 3208 |  |
| Gainful employment |  |  | 0.555 |  |  | 0.631 |  |  | 0.123 |
| No | 143172 | 50356 |  | 76020 | 25175 |  | 67152 | 25181 |  |
| Yes | 15956 | 5557 |  | 8049 | 2696 |  | 7907 | 2861 |  |
| Economic status |  |  | <0.001 |  |  | <0.001 |  |  | 0.274 |
| Very generous | 2060 | 678 |  | 1455 | 444 |  | 605 | 234 |  |
| Relatively ample | 23676 | 8045 |  | 15588 | 4994 |  | 8088 | 3051 |  |
| Basically enough | 94030 | 32620 |  | 51867 | 17075 |  | 42163 | 15545 |  |
| Tougher | 32910 | 12225 |  | 12738 | 4520 |  | 20172 | 7705 |  |
| Very difficult | 6452 | 2345 |  | 2421 | 838 |  | 4031 | 1507 |  |
| Not participating in public welfare activities |  |  | 0.007 |  |  | 0.045 |  |  | 0.008 |
| No | 72625 | 25149 |  | 36426 | 11885 |  | 36199 | 13264 |  |
| Yes | 86503 | 30764 |  | 47643 | 15986 |  | 38860 | 14778 |  |
| No external abuse |  |  | 0.569 |  |  | 0.932 |  |  | 0.361 |
| No | 7097 | 2526 |  | 3765 | 1244 |  | 3332 | 1282 |  |
| Yes | 152031 | 53387 |  | 80304 | 26627 |  | 71727 | 26760 |  |
| Non-spiritual cultural life |  |  | <0.001 |  |  | 0.004 |  |  | 0.002 |
| No | 146924 | 51225 |  | 79781 | 26327 |  | 67143 | 24898 |  |
| Yes | 12204 | 4688 |  | 4288 | 1544 |  | 7916 | 3144 |  |

**Supplement 3-table S4. The analysis of the relationship between parameters and cardiac-cerebral vascular disease by hypertension stratification.**

| Factor | All | | | Hypertension | | | | | |
| --- | --- | --- | --- | --- | --- | --- | --- | --- | --- |
|  |  |  |  | No | | | Yes | | |
|  | Without CCVD | With CCVD | **P-value** | Without CCVD | With CCVD | **P-value** | Without CCVD | With CCVD | **P-value** |
| Urban and rural |  |  | <0.001 |  |  | <0.001 |  |  | <0.001 |
| Town | 84069 | 27871 |  | 50580 | 16720 |  | 33489 | 11151 |  |
| Rural area | 75059 | 28042 |  | 50039 | 18429 |  | 25020 | 9613 |  |
| Age（years） |  |  | <0.001 |  |  | <0.001 |  |  | 0.058 |
| 60-64 | 52232 | 18681 |  | 36133 | 12825 |  | 16099 | 5856 |  |
| 65-69 | 37934 | 12789 |  | 23975 | 7966 |  | 13959 | 4823 |  |
| 70-74 | 26448 | 9312 |  | 15735 | 5430 |  | 10713 | 3882 |  |
| 75-79 | 20834 | 7297 |  | 12066 | 4189 |  | 8768 | 3108 |  |
| 80-84 | 13586 | 4840 |  | 7727 | 2867 |  | 5859 | 1973 |  |
| ≥85 | 8094 | 2994 |  | 4983 | 1872 |  | 3111 | 1122 |  |
| Sex |  |  | <0.001 |  |  | <0.001 |  |  | <0.001 |
| Female | 81370 | 30979 |  | 49266 | 18673 |  | 32104 | 12306 |  |
| Male | 77758 | 24934 |  | 51353 | 16476 |  | 26405 | 8458 |  |
| Education level |  |  | <0.001 |  |  | <0.001 |  |  | <0.001 |
| Have not attended school (including literacy classes) | 43934 | 19168 |  | 27591 | 11993 |  | 16343 | 7175 |  |
| Primary school (including private school) | 66476 | 22583 |  | 43138 | 14554 |  | 23338 | 8029 |  |
| Junior high school | 31952 | 8556 |  | 20061 | 5292 |  | 11891 | 3264 |  |
| High school/secondary school/vocational high school | 11229 | 3858 |  | 6678 | 2312 |  | 4551 | 1546 |  |
| College | 3237 | 1032 |  | 1818 | 579 |  | 1419 | 453 |  |
| Bachelor degree and above | 1739 | 583 |  | 969 | 330 |  | 770 | 253 |  |
| Marriage |  |  | <0.001 |  |  | <0.001 |  |  | <0.001 |
| Married | 116240 | 39733 |  | 74656 | 25320 |  | 41584 | 14413 |  |
| Widowed | 39278 | 14864 |  | 23337 | 8867 |  | 15941 | 5997 |  |
| Divorce | 1332 | 463 |  | 901 | 312 |  | 431 | 151 |  |
| Never married | 2278 | 853 |  | 1725 | 650 |  | 553 | 203 |  |
| Live alone |  |  | <0.001 |  |  | <0.001 |  |  | 0.004 |
| No | 138148 | 47970 |  | 87775 | 30261 |  | 50373 | 17709 |  |
| Yes | 20980 | 7943 |  | 12844 | 4888 |  | 8136 | 3055 |  |
| Smoking |  |  | 0.023 |  |  | 0.037 |  |  | 0.284 |
| No | 10871 | 3662 |  | 6576 | 2185 |  | 4295 | 1477 |  |
| Yes | 148257 | 52251 |  | 94043 | 32964 |  | 54214 | 19287 |  |
| Drinking |  |  | <0.001 |  |  | <0.001 |  |  | 0.225 |
| Never or occasionally | 156687 | 55249 |  | 98910 | 34710 |  | 57777 | 20539 |  |
| 1-2 times a week | 663 | 186 |  | 454 | 115 |  | 209 | 71 |  |
| At least 3 times a week | 1556 | 414 |  | 1087 | 277 |  | 469 | 137 |  |
| Often drunk | 222 | 64 |  | 168 | 47 |  | 54 | 17 |  |
| Sleep |  |  | <0.001 |  |  | <0.001 |  |  | <0.001 |
| That's good | 2475 | 670 |  | 1648 | 455 |  | 827 | 215 |  |
| Better | 5020 | 1511 |  | 3292 | 968 |  | 1728 | 543 |  |
| General | 148028 | 52560 |  | 93567 | 33061 |  | 54461 | 19499 |  |
| Relatively poor | 3020 | 973 |  | 1766 | 571 |  | 1254 | 402 |  |
| Very bad | 585 | 199 |  | 346 | 94 |  | 239 | 105 |  |
| Workout |  |  | <0.001 |  |  | <0.001 |  |  | <0.001 |
| Never exercise | 76957 | 28256 |  | 50576 | 18363 |  | 26381 | 9893 |  |
| Less than once | 6956 | 2497 |  | 4242 | 1507 |  | 2714 | 990 |  |
| Once or twice | 20499 | 7083 |  | 12620 | 4400 |  | 7879 | 2683 |  |
| Three to five times | 19618 | 6721 |  | 11947 | 4133 |  | 7671 | 2588 |  |
| Six times and above | 35098 | 11356 |  | 21234 | 6746 |  | 13864 | 4610 |  |
| Diabetes |  |  | 0.951 |  |  | 0.523 |  |  | 0.349 |
| No | 145270 | 51049 |  | 95369 | 33284 |  | 49901 | 17765 |  |
| Yes | 13858 | 4864 |  | 5250 | 1865 |  | 8608 | 2999 |  |
| Stomach disease |  |  | 0.173 |  |  | 0.229 |  |  | 0.517 |
| No | 130855 | 45835 |  | 82980 | 28887 |  | 47875 | 16948 |  |
| Yes | 28273 | 10078 |  | 17639 | 6262 |  | 10634 | 3816 |  |
| Medical insurance |  |  | 0.957 |  |  | 0.432 |  |  | 0.163 |
| No | 157676 | 55405 |  | 99565 | 34763 |  | 58111 | 20642 |  |
| Yes | 1452 | 508 |  | 1054 | 386 |  | 398 | 122 |  |
| Elderly people in your home who require care |  |  | 0.658 |  |  | 0.724 |  |  | 0.818 |
| No | 140396 | 49371 |  | 88554 | 30960 |  | 51842 | 18411 |  |
| Yes | 18732 | 6542 |  | 12065 | 4189 |  | 6667 | 2353 |  |
| Gainful employment |  |  | 0.555 |  |  | 0.509 |  |  | 0.903 |
| No | 143172 | 50356 |  | 89012 | 31141 |  | 54160 | 19215 |  |
| Yes | 15956 | 5557 |  | 11607 | 4008 |  | 4349 | 1549 |  |
| Economic status |  |  | <0.001 |  |  | <0.001 |  |  | 0.003 |
| Very generous | 2060 | 678 |  | 1328 | 443 |  | 732 | 235 |  |
| Relatively ample | 23676 | 8045 |  | 15238 | 5122 |  | 8438 | 2923 |  |
| Basically enough | 94030 | 32620 |  | 59672 | 20607 |  | 34358 | 12013 |  |
| Tougher | 32910 | 12225 |  | 20492 | 7566 |  | 12418 | 4659 |  |
| Very difficult | 6452 | 2345 |  | 3889 | 1411 |  | 2563 | 934 |  |
| Not participating in public welfare activities |  |  | 0.007 |  |  | 0.029 |  |  | 0.130 |
| No | 72625 | 25149 |  | 47067 | 16205 |  | 25558 | 8944 |  |
| Yes | 86503 | 30764 |  | 53552 | 18944 |  | 32951 | 11820 |  |
| No external abuse |  |  | 0.569 |  |  | 0.917 |  |  | 0.441 |
| No | 7097 | 2526 |  | 4421 | 1549 |  | 2676 | 977 |  |
| Yes | 152031 | 53387 |  | 96198 | 33600 |  | 55833 | 19787 |  |
| Non-spiritual cultural life |  |  | <0.001 |  |  | <0.001 |  |  | 0.008 |
| No | 146924 | 51225 |  | 92561 | 32048 |  | 54363 | 19177 |  |
| Yes | 12204 | 4688 |  | 8058 | 3101 |  | 4146 | 1587 |  |

**Supplement 3-table S5. The analysis of the relationship between parameters and cardiac-cerebral vascular disease by diabetes stratification.**

| Factor | All | | | Diabetes | | | | | |
| --- | --- | --- | --- | --- | --- | --- | --- | --- | --- |
|  |  |  |  | No | | | Yes | | |
|  | Without CCVD | With CCVD | **P-value** | Without CCVD | With CCVD | **P-value** | Without CCVD | With CCVD | **P-value** |
| Urban and rural |  |  | <0.001 |  |  | <0.001 |  |  | <0.001 |
| Town | 84069 | 27871 |  | 74530 | 24680 |  | 9539 | 3191 |  |
| Rural area | 75059 | 28042 |  | 70740 | 26369 |  | 4319 | 1673 |  |
| Age（years） |  |  | <0.001 |  |  | <0.001 |  |  | 0.039 |
| 60-64 | 52232 | 18681 |  | 48228 | 17191 |  | 4004 | 1490 |  |
| 65-69 | 37934 | 12789 |  | 34419 | 11580 |  | 3515 | 1209 |  |
| 70-74 | 26448 | 9312 |  | 23887 | 8413 |  | 2561 | 899 |  |
| 75-79 | 20834 | 7297 |  | 18894 | 6608 |  | 1940 | 689 |  |
| 80-84 | 13586 | 4840 |  | 12321 | 4462 |  | 1265 | 378 |  |
| ≥85 | 8094 | 2994 |  | 7521 | 2795 |  | 573 | 199 |  |
| Sex |  |  | <0.001 |  |  | <0.001 |  |  | <0.001 |
| Female | 81370 | 30979 |  | 73426 | 27959 |  | 7944 | 3020 |  |
| Male | 77758 | 24934 |  | 71844 | 23090 |  | 5914 | 1844 |  |
| Education level |  |  | <0.001 |  |  | <0.001 |  |  | <0.001 |
| Have not attended school (including literacy classes) | 43934 | 19168 |  | 40764 | 17675 |  | 3170 | 1493 |  |
| Primary school (including private school) | 66476 | 22583 |  | 61338 | 20848 |  | 5138 | 1735 |  |
| Junior high school | 31952 | 8556 |  | 28613 | 7632 |  | 3339 | 924 |  |
| High school/secondary school/vocational high school | 11229 | 3858 |  | 9834 | 3416 |  | 1395 | 442 |  |
| College | 3237 | 1032 |  | 2740 | 869 |  | 497 | 163 |  |
| Bachelor degree and above | 1739 | 583 |  | 1464 | 482 |  | 275 | 101 |  |
| Marriage |  |  | <0.001 |  |  | <0.001 |  |  | 0.435 |
| Married | 116240 | 39733 |  | 105955 | 36178 |  | 10285 | 3555 |  |
| Widowed | 39278 | 14864 |  | 35912 | 13627 |  | 3366 | 1237 |  |
| Divorce | 1332 | 463 |  | 1208 | 422 |  | 124 | 41 |  |
| Never married | 2278 | 853 |  | 2195 | 822 |  | 83 | 31 |  |
| Live alone |  |  | <0.001 |  |  | <0.001 |  |  | 0.292 |
| No | 138148 | 47970 |  | 126015 | 43740 |  | 12133 | 4230 |  |
| Yes | 20980 | 7943 |  | 19255 | 7309 |  | 1725 | 634 |  |
| Smoking |  |  | 0.023 |  |  | 0.013 |  |  | 0.779 |
| No | 10871 | 3662 |  | 9822 | 3288 |  | 1049 | 374 |  |
| Yes | 148257 | 52251 |  | 135448 | 47761 |  | 12809 | 4490 |  |
| Drinking |  |  | <0.001 |  |  | <0.001 |  |  | 0.519 |
| Never or occasionally | 156687 | 55249 |  | 142971 | 50423 |  | 13716 | 4826 |  |
| 1-2 times a week | 663 | 186 |  | 620 | 174 |  | 43 | 12 |  |
| At least 3 times a week | 1556 | 414 |  | 1465 | 390 |  | 91 | 24 |  |
| Often drunk | 222 | 64 |  | 214 | 62 |  | 8 | 2 |  |
| Sleep |  |  | <0.001 |  |  | <0.001 |  |  | 0.587 |
| That's good | 2475 | 670 |  | 2281 | 609 |  | 194 | 61 |  |
| Better | 5020 | 1511 |  | 4635 | 1371 |  | 385 | 140 |  |
| General | 148028 | 52560 |  | 135118 | 48023 |  | 12910 | 4537 |  |
| Relatively poor | 3020 | 973 |  | 2725 | 880 |  | 295 | 93 |  |
| Very bad | 585 | 199 |  | 511 | 166 |  | 74 | 33 |  |
| Workout |  |  | <0.001 |  |  | <0.001 |  |  | 0.003 |
| Never exercise | 76957 | 28256 |  | 71632 | 26287 |  | 5325 | 1969 |  |
| Less than once | 6956 | 2497 |  | 6349 | 2274 |  | 607 | 223 |  |
| Once or twice | 20499 | 7083 |  | 18717 | 6440 |  | 1782 | 643 |  |
| Three to five times | 19618 | 6721 |  | 17679 | 6023 |  | 1939 | 698 |  |
| Six times and above | 35098 | 11356 |  | 30893 | 10025 |  | 4205 | 1331 |  |
| Hypertension |  |  | 0.121 |  |  | 0.066 |  |  | 0.571 |
| No | 100619 | 35149 |  | 95369 | 33284 |  | 5250 | 1865 |  |
| Yes | 58509 | 20764 |  | 49901 | 17765 |  | 8608 | 2999 |  |
| Stomach disease |  |  | 0.173 |  |  | 0.635 |  |  | 0.201 |
| No | 130855 | 45835 |  | 119218 | 41765 |  | 11637 | 4070 |  |
| Yes | 28273 | 10078 |  | 26052 | 9284 |  | 2221 | 794 |  |
| Medical insurance |  |  | 0.957 |  |  | 0.748 |  |  | 0.273 |
| No | 157676 | 55405 |  | 143893 | 50574 |  | 13783 | 4831 |  |
| Yes | 1452 | 508 |  | 1377 | 475 |  | 75 | 33 |  |
| Elderly people in your home who require care |  |  | 0.658 |  |  | 0.725 |  |  | 0.716 |
| No | 140396 | 49371 |  | 128167 | 45069 |  | 12229 | 4302 |  |
| Yes | 18732 | 6542 |  | 17103 | 5980 |  | 1629 | 562 |  |
| Gainful employment |  |  | 0.555 |  |  | 0.624 |  |  | 0.649 |
| No | 143172 | 50356 |  | 130165 | 45781 |  | 13007 | 4575 |  |
| Yes | 15956 | 5557 |  | 15105 | 5268 |  | 851 | 289 |  |
| Economic status |  |  | <0.001 |  |  | <0.001 |  |  | 0.018 |
| Very generous | 2060 | 678 |  | 1844 | 618 |  | 216 | 60 |  |
| Relatively ample | 23676 | 8045 |  | 21512 | 7303 |  | 2164 | 742 |  |
| Basically enough | 94030 | 32620 |  | 85841 | 29816 |  | 8189 | 2804 |  |
| Tougher | 32910 | 12225 |  | 30241 | 11188 |  | 2669 | 1037 |  |
| Very difficult | 6452 | 2345 |  | 5832 | 2124 |  | 620 | 221 |  |
| Not participating in public welfare activities |  |  | 0.007 |  |  | 0.004 |  |  | 0.906 |
| No | 72625 | 25149 |  | 66906 | 23137 |  | 5719 | 2012 |  |
| Yes | 86503 | 30764 |  | 78364 | 27912 |  | 8139 | 2852 |  |
| No external abuse |  |  | 0.569 |  |  | 0.608 |  |  | 0.793 |
| No | 7097 | 2526 |  | 6392 | 2274 |  | 705 | 252 |  |
| Yes | 152031 | 53387 |  | 138878 | 48775 |  | 13153 | 4612 |  |
| Non-spiritual cultural life |  |  | <0.001 |  |  | <0.001 |  |  | 0.835 |
| No | 146924 | 51225 |  | 133891 | 46655 |  | 13033 | 4570 |  |
| Yes | 12204 | 4688 |  | 11379 | 4394 |  | 825 | 294 |  |
